# Butterfly Diversity and Community Dynamics in the Central Himalayas: Species Composition, Richness, Abundance, and Seasonal Variation of Butterflies (Lepidoptera: Papilionoidea) in Bhorletar, Nepal

**DOI:** 10.1002/ece3.70612

**Published:** 2024-12-17

**Authors:** Sajan KC, Anisha Sapkota

**Affiliations:** ^1^ Institute of Agriculture and Animal Science Tribhuvan University Sundarbazar Gandaki Province Nepal; ^2^ Agriculture and Forestry University Bharatpur Bagmati Province Nepal

**Keywords:** biodiversity, conservation, ecology, habitat, natural history

## Abstract

Butterflies are among the most effective bioindicators of climate change; however, their diversity in many rural areas of the Central Himalayas remains understudied. This study provides an assessment of butterfly diversity in the foothills of Bhorletar, Madhya Nepal Municipality, Lamjung District, Nepal, within an elevation range of 420–600 m. Conducted between July 2019 and January 2021, the survey involved opportunistic observations and photography of adult butterflies in their natural habitats, with sampling occurring six times each month. The study aimed to investigate the species composition, richness, and abundance of butterflies across the survey period and identify seasonal changes in species composition and richness. A total of 94,009 individuals across 226 species, 129 genera, and six families were documented. During this study, *Halpe arcuata* Evans, 1937 and *Hasora taminatus bhavara* Fruhstorfer, 1911 were recorded for the first time in Nepal. Additionally, *Halpe filda* Evans, 1949 and *Ctenoptilum vasava vasava* (Moore, [1866]) were recorded for only the second and third times, respectively, in Nepal, following a gap of approximately three decades. The most abundant species was *Pieris canidia indica* Evans, 1926 (Relative Abundance [RA] 2.55%), followed by *Pseudozizeeria maha maha* (Kollar, [1844]) (RA 2.13%). Species richness showed an annual bimodal distribution, peaking in April (180 species) and August (161 species), while the lowest richness was observed in January and February, with 68 and 75 species, respectively. Diversity indices included a Shannon–Wiener index of 4.71, Pielou's *J* index of 0.87, an effective number of species of 111.24, and Margalef's richness index of 19.65, indicating high species diversity with a well‐balanced mix of species evenness and richness. This study offers the first peer‐reviewed checklist of butterflies from Bhorletar, providing crucial baseline data for future research and conservation efforts, and highlights the remarkable seasonal and species diversity within the region.

## Graphical abstract

This study presents an assessment of butterfly diversity in the foothills of Bhorletar, Lamjung District, Nepal, documenting 226 species across 129 genera and six families. The research reveals a bimodal pattern of species richness, peaking in April and August, with significant implications for conservation and management strategies. The findings highlight significant seasonal and species diversity, providing crucial baseline data for future research and conservation efforts.

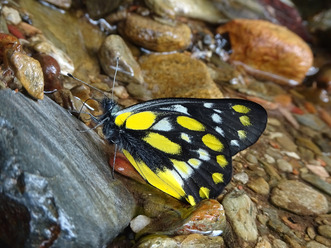

## 1 Introduction

Butterflies are among the most effective bioindicators of environmental change due to several key factors. Their large size and vibrant coloration make them relatively easy to identify and study (Brown Jr. 1991). Their ectothermic physiology makes them highly sensitive to shifts in climate conditions affecting their behaviors and life cycles (Guedes et al. 2000; Roy and Sparks 2000; Pateman et al. 2012). Their mobility allows them to rapidly respond to environmental changes, making them valuable for monitoring ecological shifts (Cormont et al. 2011). Studying butterfly populations offers critical insights into the impacts of climate change on biodiversity and ecosystem health (Hill et al. 2021) given their close associations with specific environmental conditions and host plants (Van Nouhuys and Hanski 2005). Thus, establishing clear, quantitative long‐term data on their population status, similar to the well‐established North American Butterfly Monitoring Network and the European Butterfly Monitoring Scheme (eBMS), could be highly beneficial for conservation efforts.

Nepal, a small country situated in the Central Himalayas, spans 147,181 km^2^ between 26°22' and 30°27' N latitude and 80°04' and 88°12' E longitude (Karki et al. 2016). Its diverse terrain encompasses a vast range of elevations, from 60 m (Dhital 2015) to 8849 m (Burtscher and Viscor 2022). This elevational variation, combined with its geographic location, creates a diverse range of climates, including tropical and subtropical in the southern plains, temperate in the mid‐hills, and subalpine to nival in the northern mountains (Barrueto et al. 2017). Consequently, Nepal hosts an impressive array of biodiversity, encompassing over 100 distinct ecosystems (Paudel, Bhattarai, and Kindlmann 2012). Nepal's small size and diverse ecosystems make it an ideal location for ecological studies. However, the documentation of biodiversity in Nepal, particularly insect diversity, is still in its preliminary stages. In many rural landscapes of Nepal, baseline data on butterfly diversity and abundance are notably scarce. Numerous reports have consistently highlighted an alarming decline in insect abundance and diversity worldwide, attributed to the current climate change scenario (Fonseca 2009; Vanbergen and Initiative Insect Pollinators 2013; Hallmann et al. 2017; Sánchez‐Bayo and Wyckhuys 2019; Seibold et al. 2019). This trend is particularly concerning for Nepal's biodiversity, as the Himalayan region is extremely vulnerable to climate change impacts (Kreft, Eckstein, and Melchior 2016; Ojha et al. 2016; Mainali and Pricope 2017), including the Central Himalayas (Pant et al. 2018). The region faces escalating threats from forest fires, landslides, extreme temperature events, pollution, and deforestation, which collectively imperil its biodiversity (Bhattacharjee et al. 2017; Upreti and Upreti 2002).

The cataloging of butterflies in Nepal dates to Gough (1935), who reported 150 species. Smith (2010) compiled a comprehensive catalog of 661 butterfly species from Nepal; their general distribution and images of spread specimens are presented in Smith (2006, 2011a). Building on Smith's (2010) checklist, the update of Van der Poel and Smetacek (2022) included recently discovered butterfly records, enhancing the accuracy and comprehensiveness of the checklist. While Smith (2006) divided Nepal into four ecological zones: Western, Eastern, Central, and Kathmandu Valley, Van der Poel and Smetacek (2022) refined this classification into 10 ecological zones—West Terai, East Terai, West, Karnali, East, Gandaki, Mustang‐Manang, Pokhara, Kathmandu Valley, and Bagmati—to improve accuracy and better reflect the unique butterfly diversity in each zone. Although Colin Smith conducted extensive surveys across many hill regions of Nepal, including the study area (his exclusive data were not published), his work primarily focused on creating checklists and catalogs. While he assigned rarity statuses to species based on their abundance, indicating some level of quantification, his work lacked in‐depth statistical analyses. Recent studies on butterfly diversity in the central mid‐hills of Nepal, such as by Miya et al. (2021) and Subedi et al. (2021), have effectively utilized diversity indices and statistical methods to shed light on the country's butterfly diversity, showcasing the value of quantitative approaches in understanding this complex ecosystem. However, significant knowledge gaps persist in the Central Himalayas concerning butterfly diversity and seasonal dynamics (Pandey et al. 2017; Dewan et al. 2024).

Although the species data from this study were shared with Van der Poel and Smetacek (2022) and anomalous records were presented independently in KC and Sapkota (2022), a detailed checklist of butterflies specific to the study area, along with diversity indices, was deemed essential for future researchers working in this or the neighboring areas. The objective of this paper is to present a butterfly checklist based on our 18‐month survey, highlight seasonal variations in species composition and richness, and provide a quantitative assessment of the overall diversity. Moreover, the remarkable butterfly diversity observed in this relatively small study area provides valuable insights into the broader butterfly diversity of the Gandaki region or the Central Himalayas.

## 2 Study Area

**FIGURE 1:**
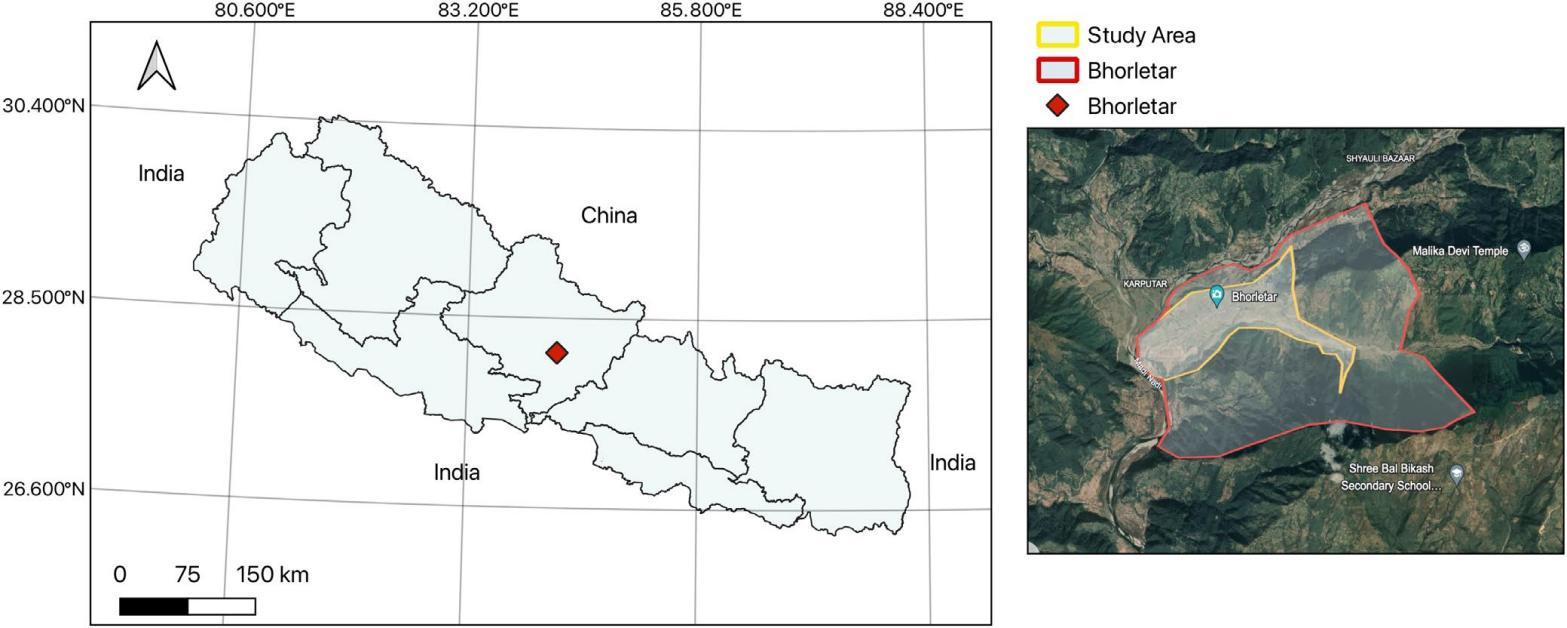
Map of Nepal (left) with the study area marked, and an inset map (right) showing the survey area in detail.

Bhorletar, a small town in Ward Number 6 of Madhya Nepal Municipality, Lamjung District is in the central mid‐hills of Nepal at 28°09'20.1" N, 84°14'15.1" E (Figure 1); it spans approximately 16 km^2^ with elevations ranging from about 420 to 600 m in the foothills (tropical climate) to about 1100 m (subtropical climate) in the high hills (measured using Google Earth 2024 (https://earth.google.com)). According to Smith's classification (Smith 2006), Bhorletar falls within the Central butterfly zone, while Van der Poel and Smetacek (2022) categorized it within the Gandaki Zone. Located 45 km west of the district headquarters, Besisahar, the region experiences a tropical to subtropical climate. The mean annual temperatures in 2019 and 2020 were 18.76°C and 18.29°C, respectively, with an average daily precipitation of 5.27 mm across both years; the average relative humidity was 66% in 2019 and 70% in 2020 (NASA Power 2024). The year can be divided into the following seasons: Spring/Pre‐monsoon (March–May), Summer/Monsoon (June–August), Autumn/Post‐monsoon (September–November), and Winter (December–February) (Mäkelä, Shrestha, and Karki 2014).

Bhorletar is located between two perennial rivers, Midim Khola to the north and Madi Nadi to the west, the latter being a tributary of the Gandaki River (Khanal 2004); additionally, Pisti Khola flows through the ward's center. Dense, mixed forests, characterized by deciduous and evergreen trees such as *Castanopsis indica* (Roxb. ex Lindl.) A.DC. (Fagaceae), *Shorea robusta* Gaertn. (Dipterocarpaceae), and *Schima wallichii* (DC.) Korth. (Theaceae), line the riverbanks, especially along Pisti Khola; small brooks and streams meander through these forests, attracting numerous butterflies during the nondry months (April–October); the local communities utilize these forests for harvesting firewood, timber, and vegetable ferns; however, forest fires pose a significant threat during the dry season (personal observation). The local economy relies primarily on subsistence farming, with paddy and maize as the major summer crops, followed by potatoes, mustard, and cole crops in winter. While urbanization is occurring gradually, deforestation and natural calamities such as landslides, exacerbated by unsustainable road construction and uncontrolled forest fires, pose significant threats to natural habitats.

## 3 Material and Methods

**FIGURE 2:**
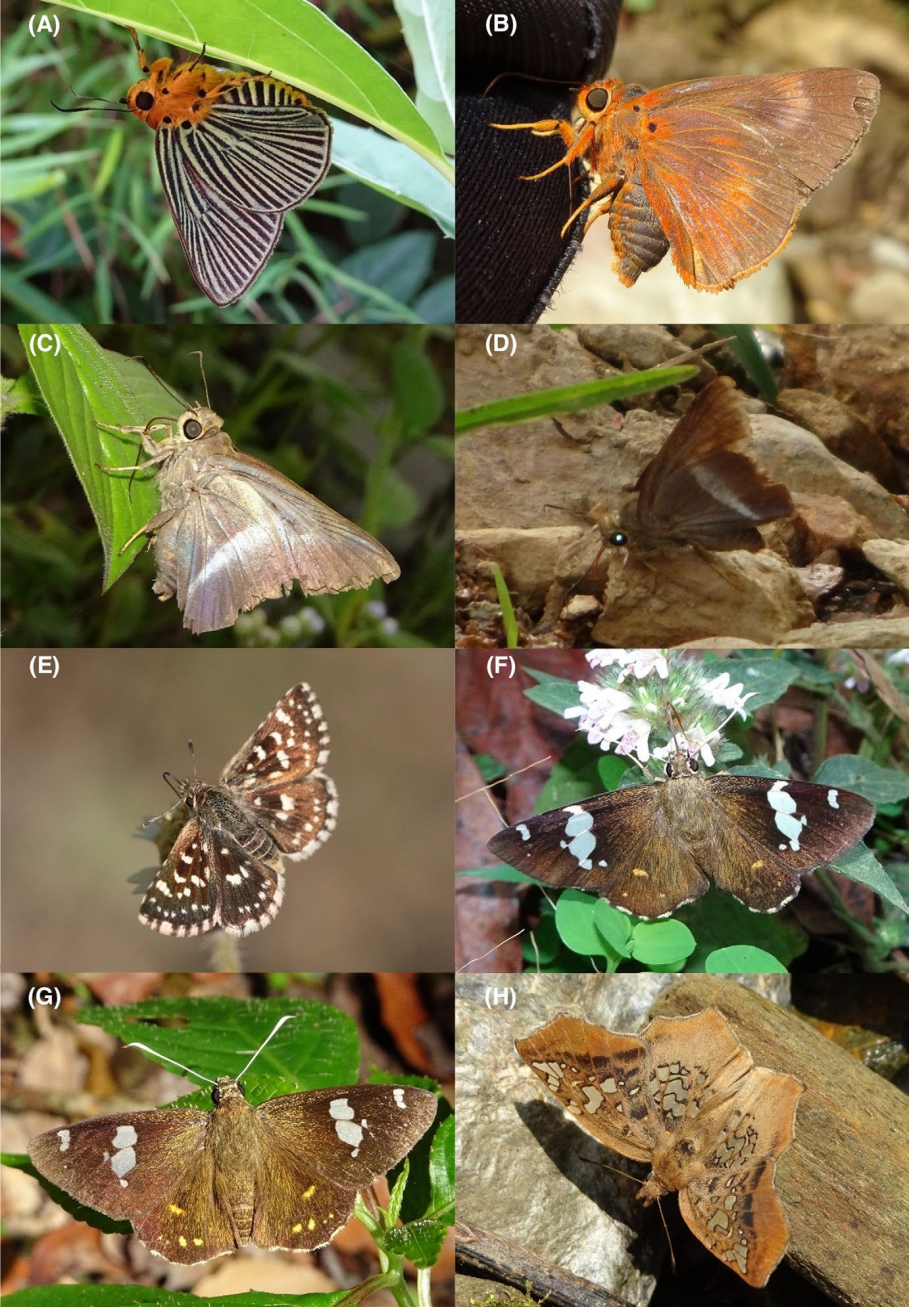
Hesperiidae of Bhorletar. (A) *Burara amara* (Moore, [1866])—Small Green Awlet; (B) *Burara oedipodea belesis* (Mabille, 1876)—Branded Orange Awlet; (C) *Hasora taminatus bhavara* Fruhstorfer, 1911—White‐Banded Awl; (D) Same individual as 2C; (E) *Spialia galba galba* (Fabricius, 1793)—Indian Skipper; (F) *Celaenorrhinus leucocera* (Kollar, [1844])—Common Spotted Flat; (G) *Celaenorrhinus putra putra* (Moore, [1866])—Bengal Spotted Flat; (H) *Ctenoptilum vasava vasava* (Moore, [1866])—Tawny Angle.

**FIGURE 3:**
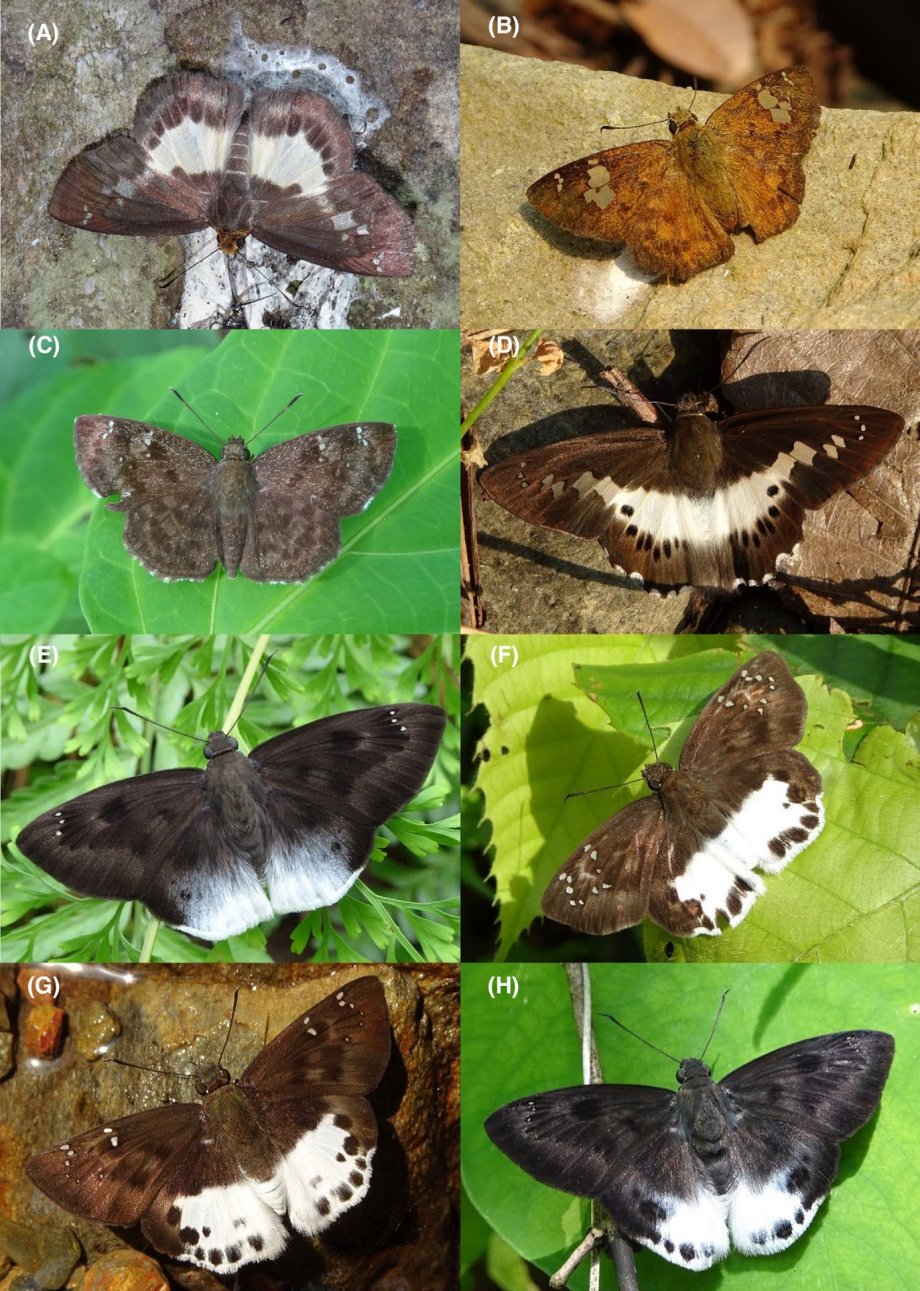
Hesperiidae of Bhorletar. (A) *Gerosis phisara phisara* (Moore, 1884)—Dusky Yellow‐Breasted Flat; (B) *Pseudocoladenia fatih* (Kollar, [1844])—Fulvous Pied Flat; (C) *Sarangesa dasahara dasahara* (Moore, [1866])—Common Small Flat; (D) *Seseria dohertyi dohertyi* Watson, 1893—Himalayan White Flat; (E) *Tagiades gana athos* Plötz, 1884—Suffused Snow Flat; (F) *Tagiades litigiosa litigiosa* Fruhstorfer, 1910—Water Snow Flat; (G) *Tagiades menaka menaka* (Moore, [1866])—Spotted Snow Flat; (H) *Tagiades parra gala* Evans, 1949—Large Snow Flat.

**FIGURE 4:**
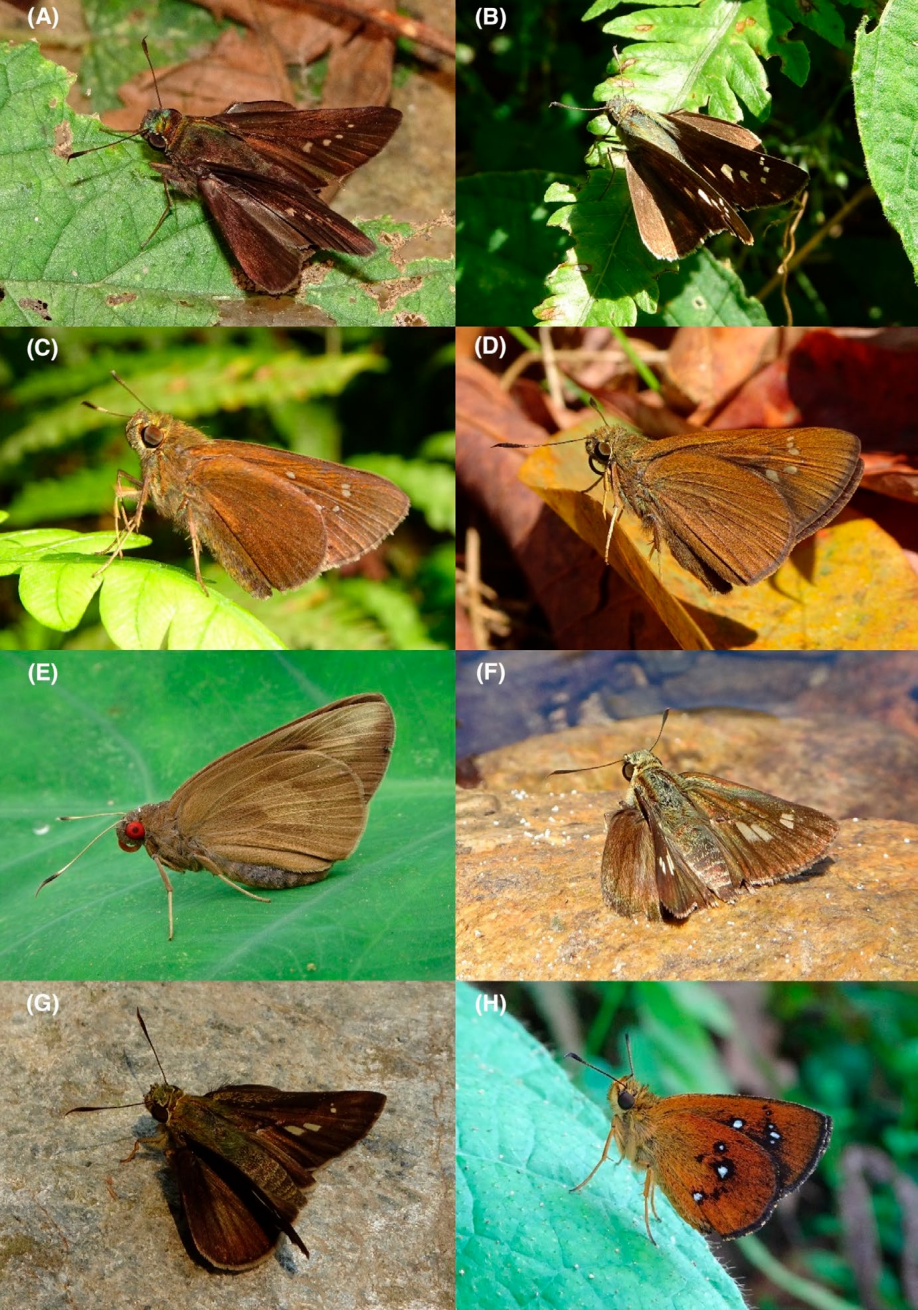
Hesperiidae of Bhorletar. (A) *Baoris farri farri* (Moore, 1878)—Paintbrush Swift; (B) *Borbo cinnara* (Wallace, 1866)—Rice Swift; (C) *Caltoris cahira austeni* (Moore, [1884])—Colon Swift; (D) *Caltoris kumara moorei* (Evans, 1926)—Blank Swift; (E) *Erionota torus* Evans, 1941—Sikkim Palm Red Eye; (F) *Halpe arcuata* Evans, 1937—Overlapped Ace; (G) *Halpe filda* Evans, 1949—Elwes' Ace; (H) *Iambrix salsala salsala* (Moore, [1866])—Chestnut Bob.

**FIGURE 5:**
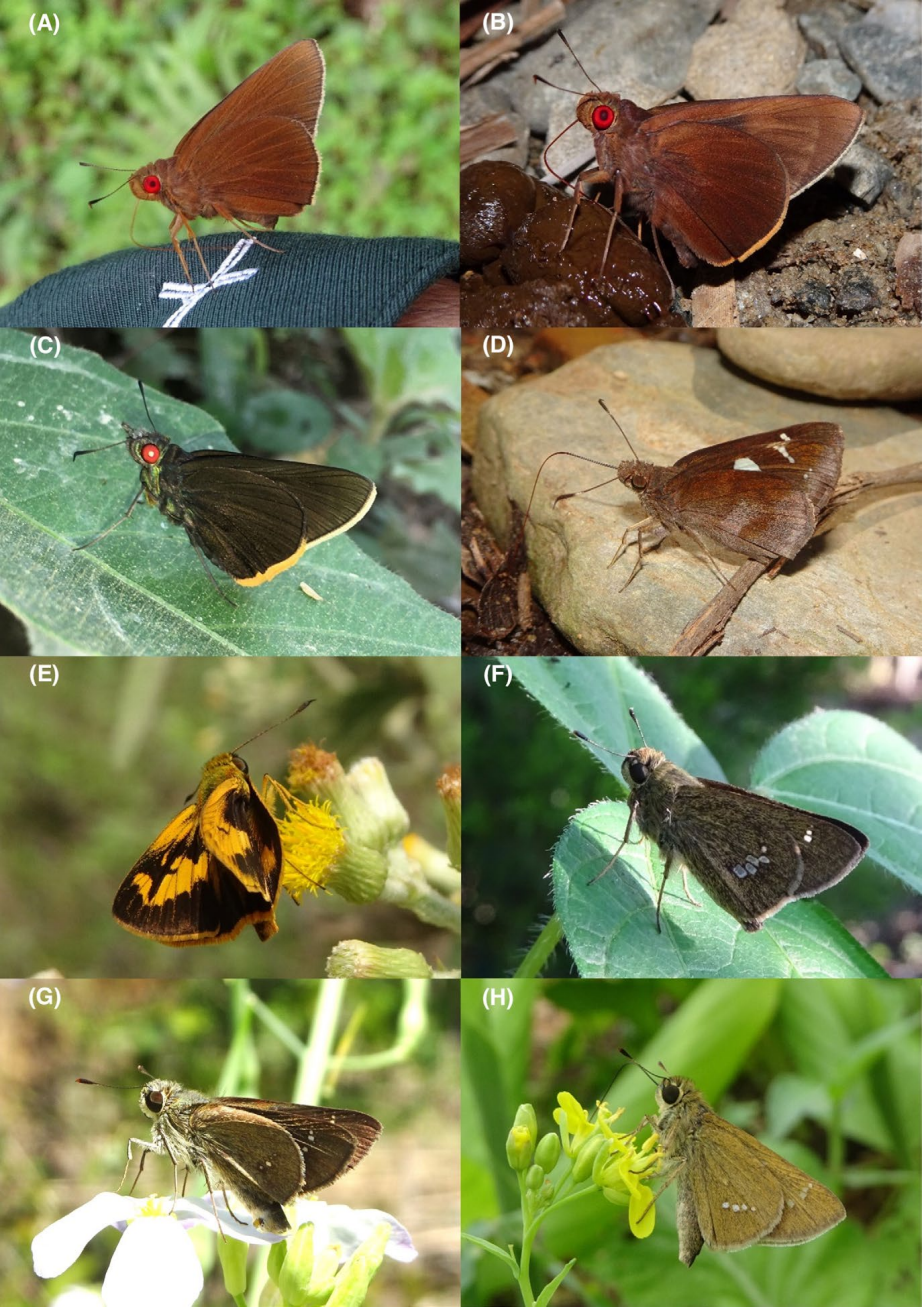
Hesperiidae of Bhorletar. (A) *Matapa aria* (Moore, [1866])—Common Red Eye; (B) *Matapa druna* (Moore, [1866])—Grey‐Brand Red Eye; (C) *Matapa sasivarna* (Moore, [1866])—Black‐Veined Red Eye; (D) *Notocrypta curvifascia curvifascia* (C. & R. Felder, 1862)—Restricted Demon; (E) *Oriens goloides* (Moore, [1881])—Ceylon Dartlet; (F) *Parnara apostata debdasi* Chiba & Eliot, 1991—Sumatran Swift; (G) *Parnara bada bada* (Moore, 1878)—Ceylon Swift; (H) *Parnara guttatus mangala* (Moore, [1866])—Straight Swift.

**FIGURE 6:**
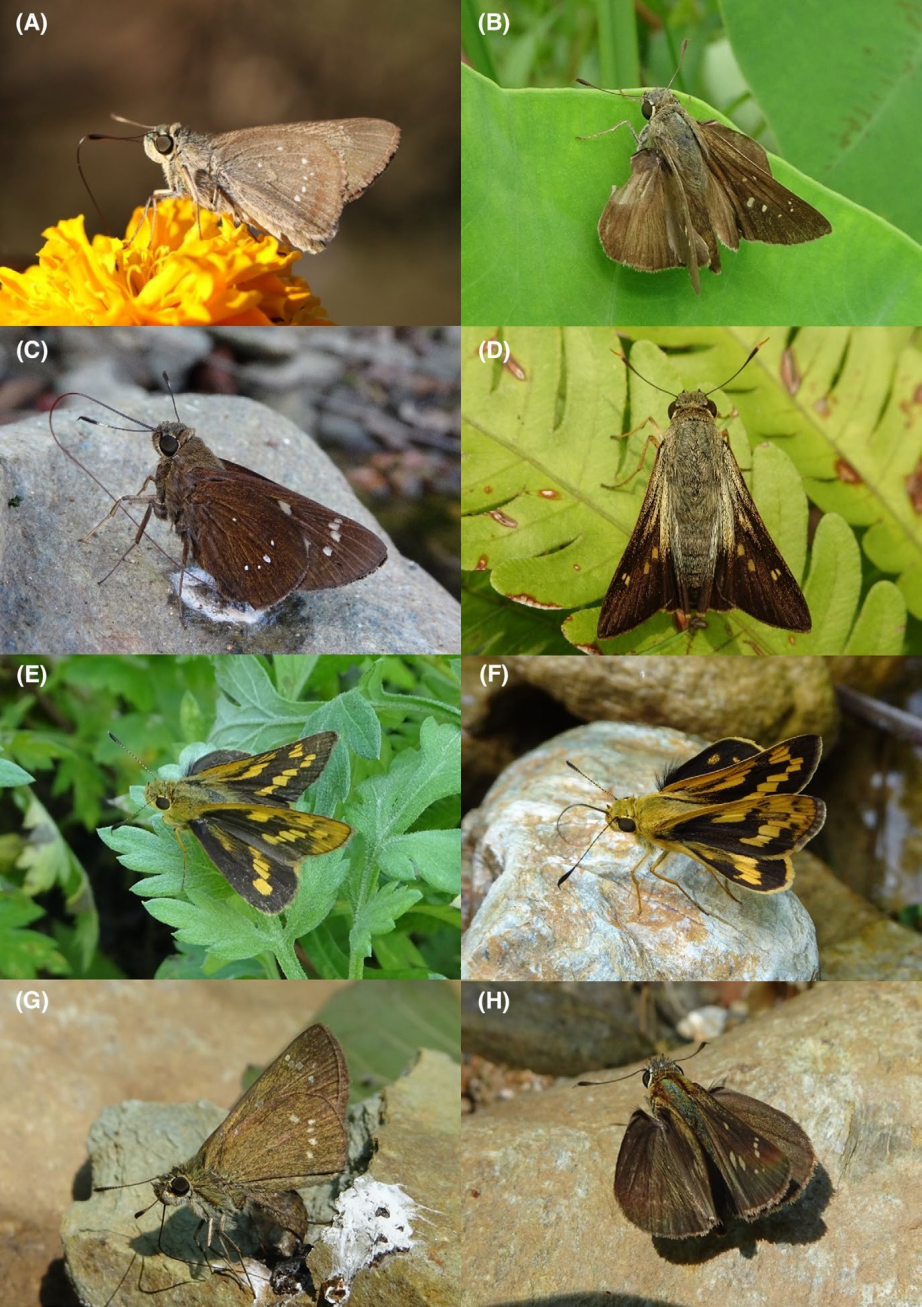
Hesperiidae of Bhorletar. (A) *Pelopidas mathias mathias* (Fabricius, 1798)—Small Branded Swift; (B) *Pelopidas agna agna* (Moore, [1866])—Little Branded Swift; (C) *Pelopidas assamensis* (de Nicéville, 1882)—Great Swift; (D) *Pithauria stramineipennis stramineipennis* Wood‐Mason & de Nicéville, [1887]—Light Straw Ace; (E) *Potanthus pallida* (Evans, 1932)—Pale Dart; (F) *Potanthus pseudomaesa clio* (Evans, 1932)—Indian Dart; (G) *Pseudoborbo bevani* (Moore, 1878)—Bevan's Swift; (H) Same individual as 6G, dorsal side.

**FIGURE 7:**
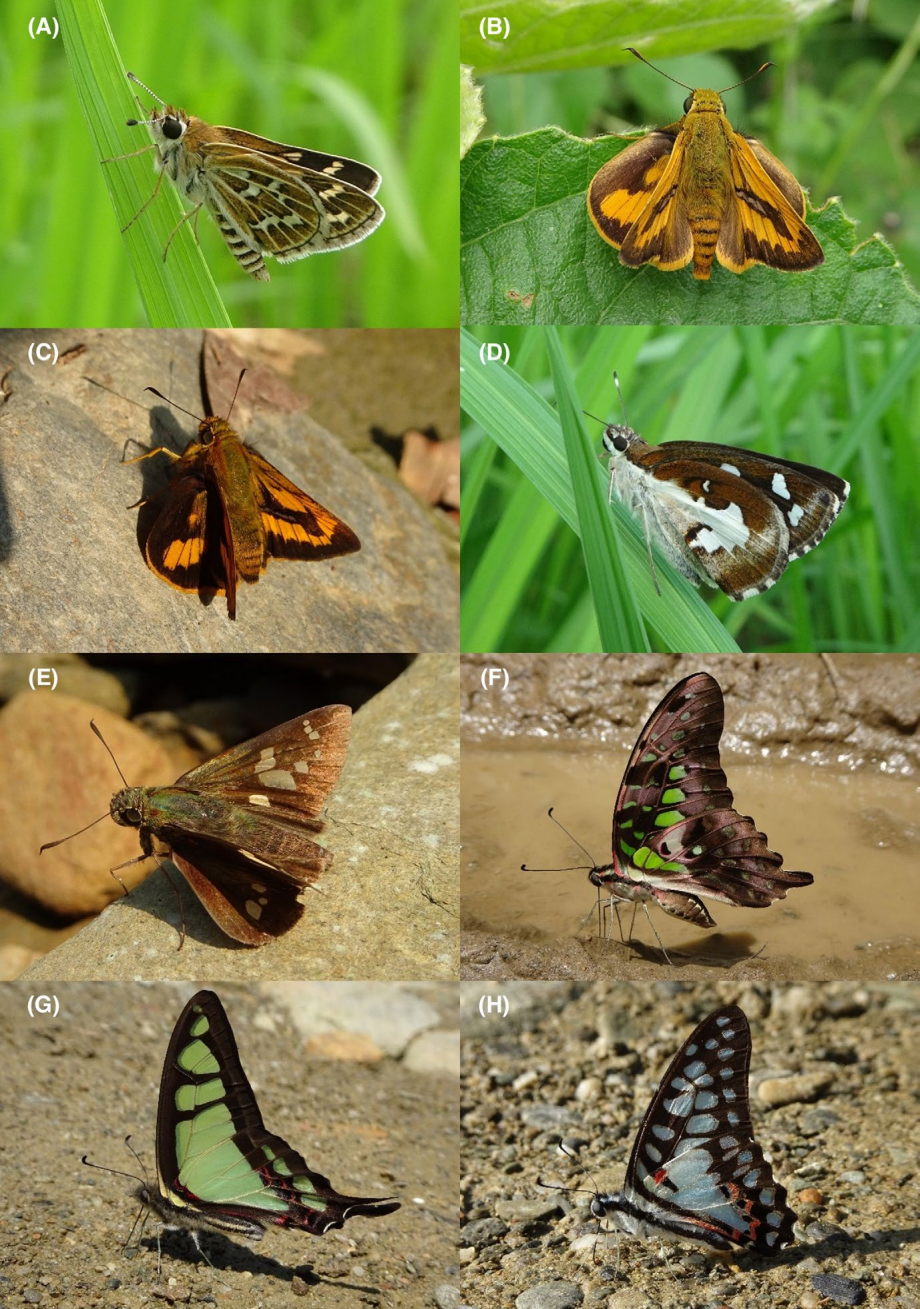
Hesperiidae and Papilionidae of Bhorletar. (A) *Taractrocera maevius sagara* (Moore, [1866])—Common Grass Dart; (B) *Telicota bambusae* (Moore, 1878)—Dark Palm Dart; (C) *Telicota ohara jix* Evans, 1949—Plotz's Palm Dart; (D) *Udaspes folus* (Cramer, [1775])—Grass Demon; (E) *Zenonoida discreta discreta* (Elwes & Edwards, 1897)—Himalayan Swift; (F) *Graphium agamemnon agamemnon* (Linnaeus, 1758)—Tailed Jay; (G) *Graphium cloanthus cloanthus* (Westwood, 1841)—Glassy Bluebottle; (H) *Graphium doson axionides* (Page & Treadaway, 2014)—Common Jay.

**FIGURE 8:**
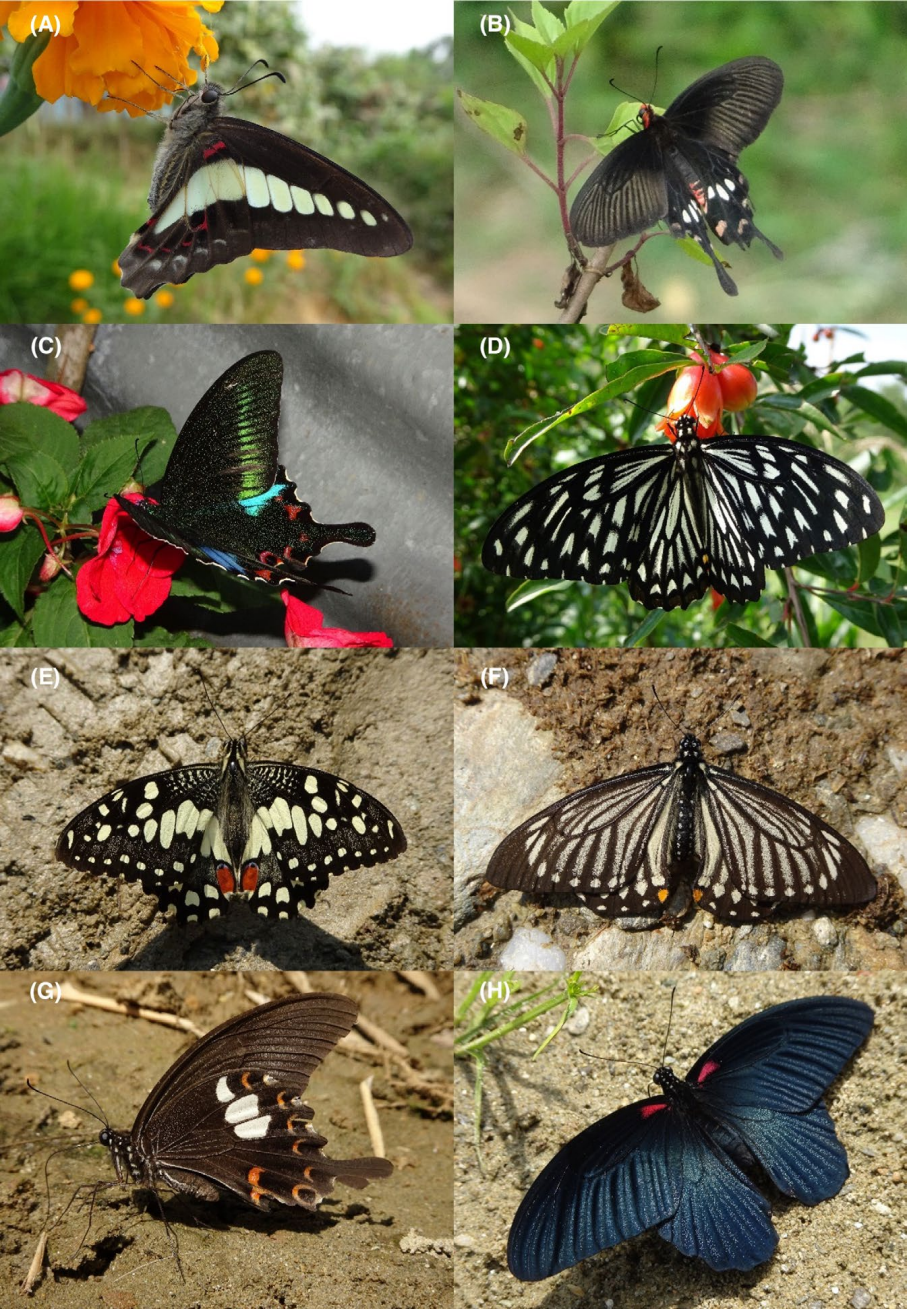
Papilionidae of Bhorletar. (A) *Graphium sarpedon sarpedon* (Linnaeus, 1758)—Common Bluebottle; (B) *Pachliopta aristolochiae aristolochiae* (Fabricius, 1775)—Common Rose; (C) *Papilio bianor ganesa* Moore, 1842—Common Peacock; (D) *Papilio clytia clytia* Linnaeus, 1758—Common Mime; (E) *Papilio demoleus demoleus* Linnaeus, 1758—Lime Swallowtail; (F) *Papilio epycides epycides* Hewitson, 1864—Lesser Mime; (G) *Papilio helenus helenus* Linnaeus, 1758—Red Helen; (H) *Papilio memnon agenor* Linnaeus, 1758—Great Mormon.

**FIGURE 9:**
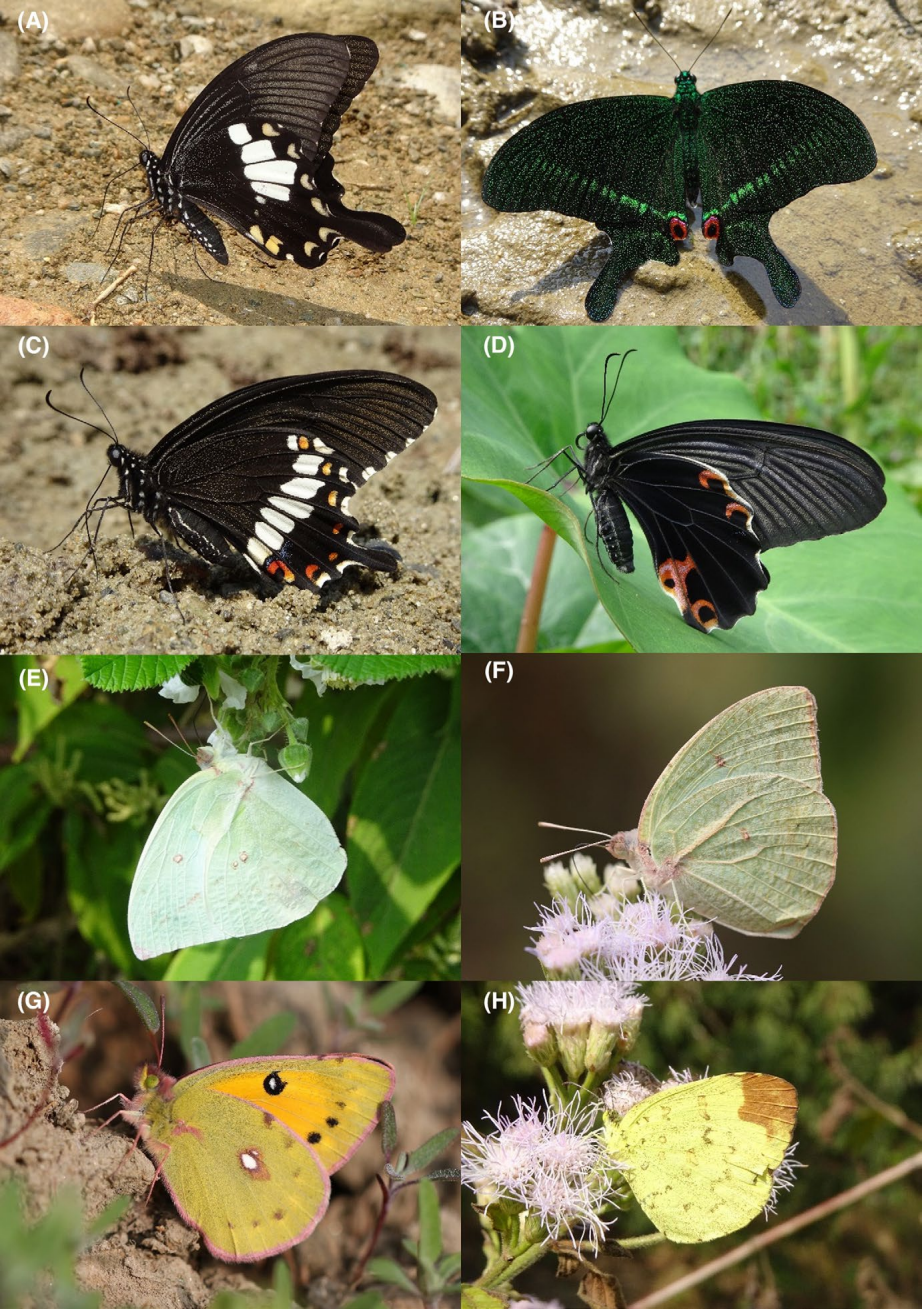
Papilionidae and Pieridae of Bhorletar. (A) *Papilio nephelus chaon* Westwood, 1844—Yellow Helen; (B) *Papilio paris paris* Linnaeus, 1758—Paris Peacock; (C) *Papilio polytes romulus* Cramer, [1775]—Common Mormon; (D) *Papilio protenor euprotenor* Fruhstorfer, 1908—Spangle; (E) *Catopsilia pomona pomona* (Fabricius, 1775)—Common Emigrant; (F) *Catopsilia pyranthe pyranthe* (Linnaeus, 1758)—Mottled Emigrant; (G) *Colias fieldii fieldii* Ménétriés, 1855—Dark Clouded Yellow; (H) *Eurema blanda silhetana* (Wallace, 1867)—Three‐Spot Grass Yellow.

**FIGURE 10:**
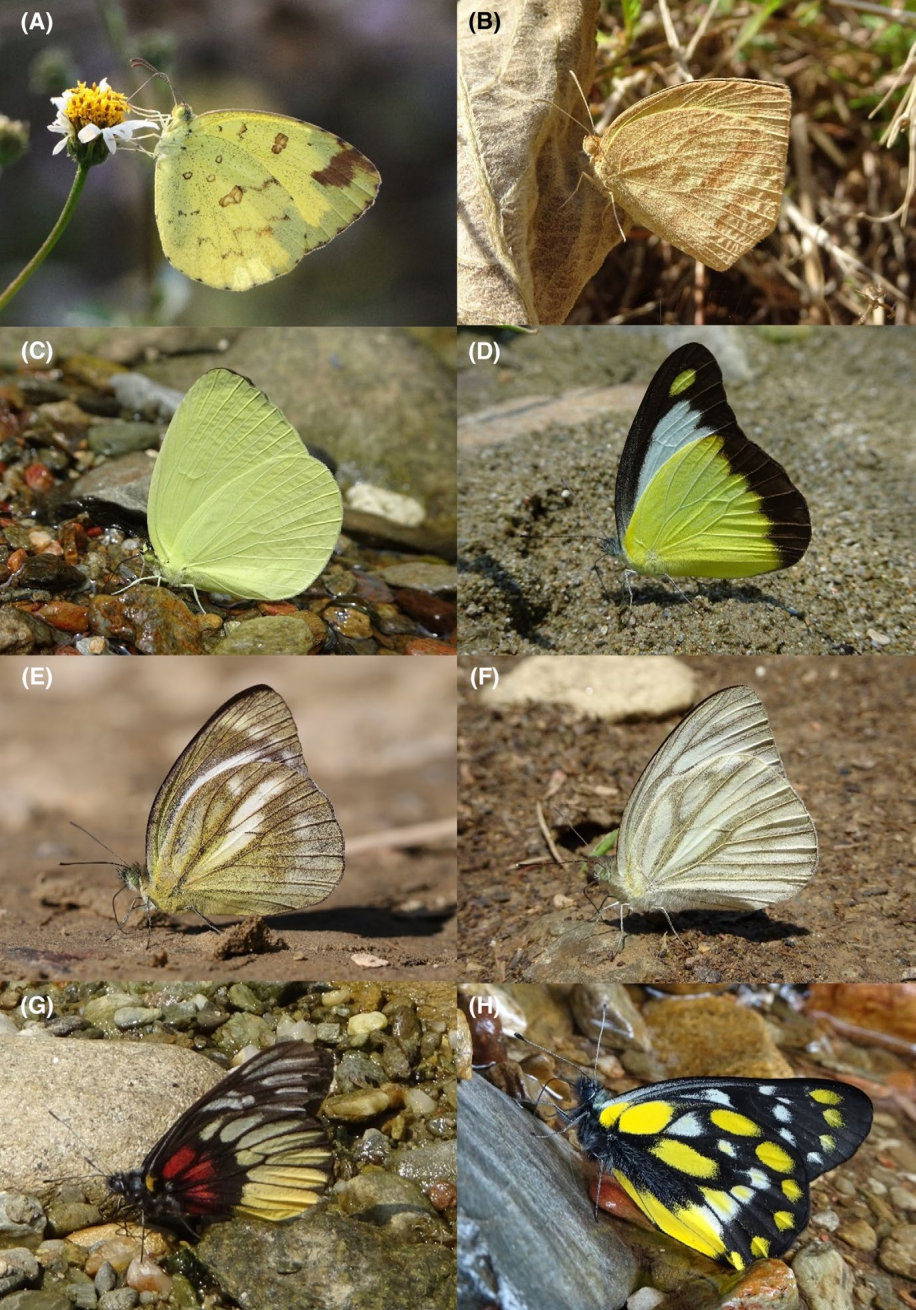
Pieridae of Bhorletar. (A) *H. Eurema hecabe hecabe* (Linnaeus, 1758)—Common Grass Yellow; (B) *Eurema laeta laeta* (Boisduval, 1836)—Spotless Grass Yellow; (C) *Gandaca harina assamica* Moore, [1906]—Tree Yellow; (D) *Appias lyncida eleonora* (Boisduval, 1836)—Chocolate Albatross; (E) *Cepora nadina nadina* (Lucas, 1852)—Lesser Gull; (F) *Cepora nerissa phryne* (Fabricius, 1775)—Common Gull; (G) *Delias acalis pyramus* (Wallace, 1867)—Red‐Breast Jezebel; (H) *Delias belladonna horsfieldi* (Gray, 1831)—Hill Jezebel.

**FIGURE 11:**
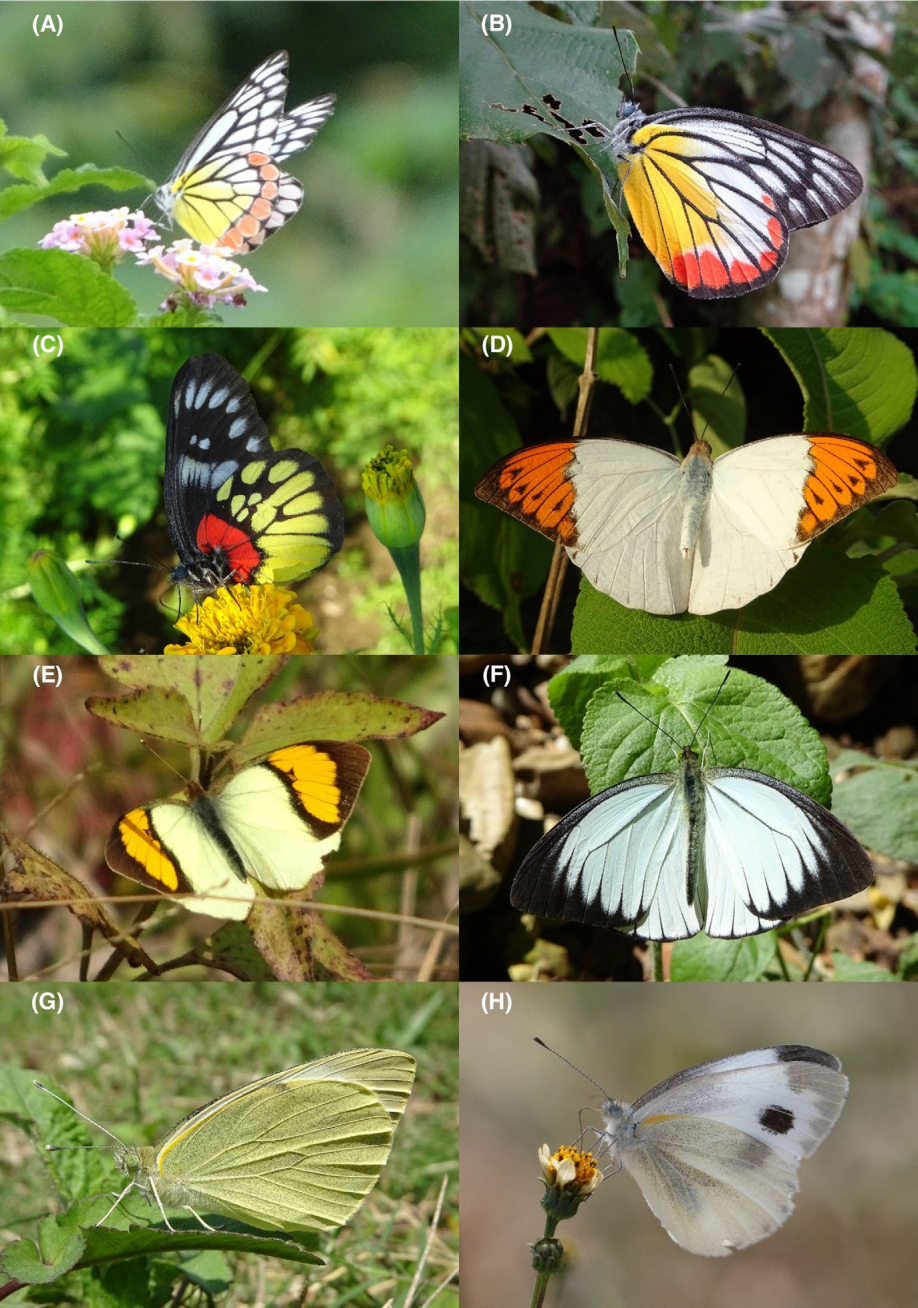
Pieridae of Bhorletar. (A) *Delias eucharis* (Drury, 1773)—Common Jezebel from Pokhara, Kaski; (B) *Delias hyparete indica* (Wallace, 1867)—Painted Jezebel; (C) *Delias pasithoe dione* (Drury, [1773])—Red‐Base Jezebel; (D) *Hebomoia glaucippe glaucippe* (Linnaeus, 1758)—Great Orange Tip; (E) *Ixias pyrene latifasciata* (Fabricius, 1777)—Yellow Orange Tip; (F) *Pareronia avatar* (Moore, [1858]) Pale Wanderer; (G) *Pieris brassicae nepalensis* Gray, 1846—Large Cabbage White; (H) *Pieris canidia indica* Evans, 1926—Indian Cabbage White.

**FIGURE 12:**
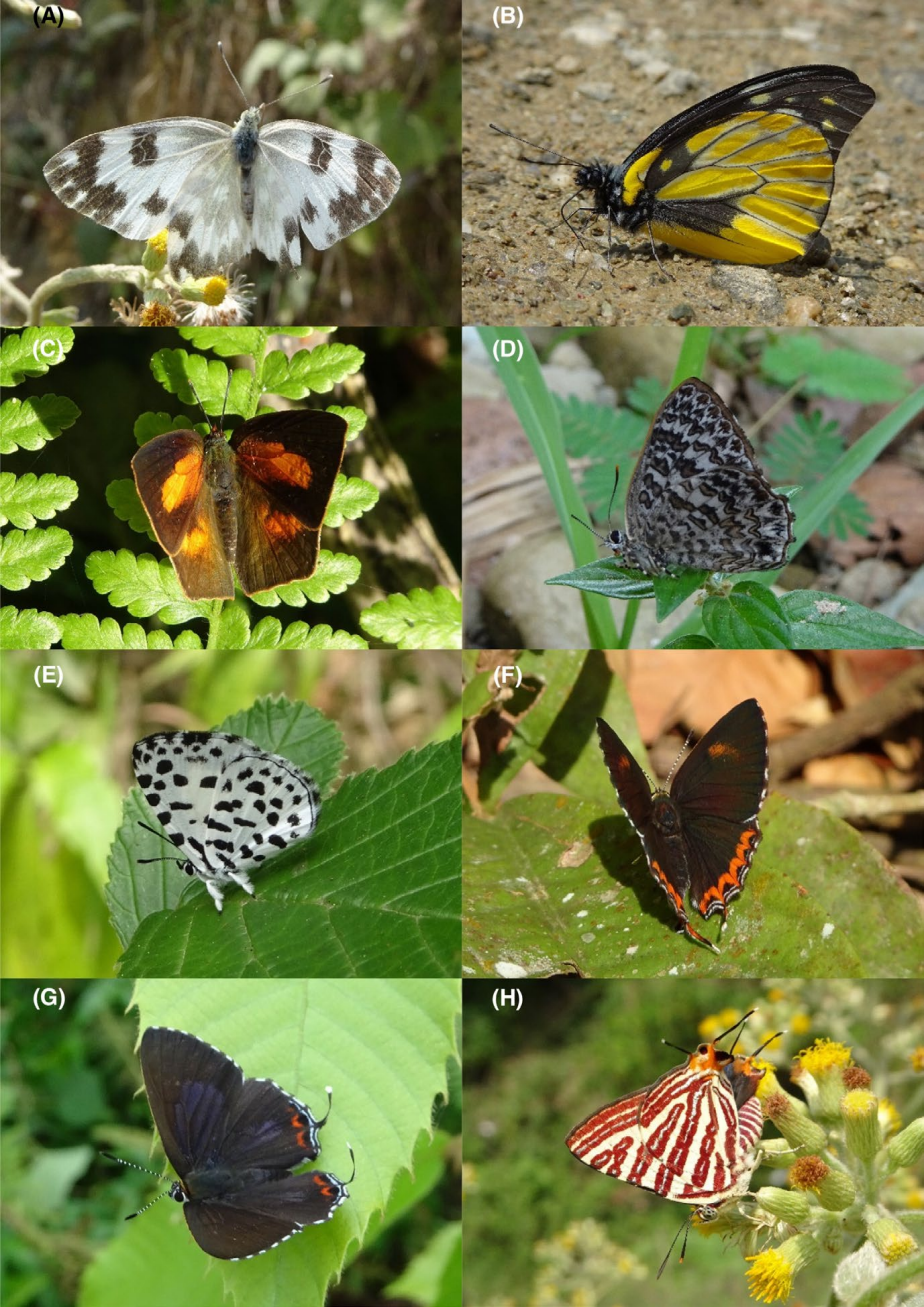
Pieridae and Lycaenidae of Bhorletar. (A) *Pontia daplidice moorei* (Röber, [1907])—Bath White; (B) *Prioneris thestylis thestylis* (Doubleday, 1842)—Spotted Sawtooth; (C) *Curetis bulis bulis* (Westwood, 1852)—Bright Sunbeam; (D) *Poritia hewitsoni hewitsoni* Moore, [1866]—Common Gem; (E) *Taraka hamada mendesia* Fruhstorfer, 1918 – Forest Pierrot; (F) *Heliophorus epicles latilimbata* Eliot, 1963—Purple Sapphire; (G) *Heliophorus indicus* (Fruhstorfer, 1908)—Indian Purple Sapphire; (H) *Spindasis lohita himalayanus* (Moore, 1884)—Long‐Banded Silverline.

**FIGURE 13:**
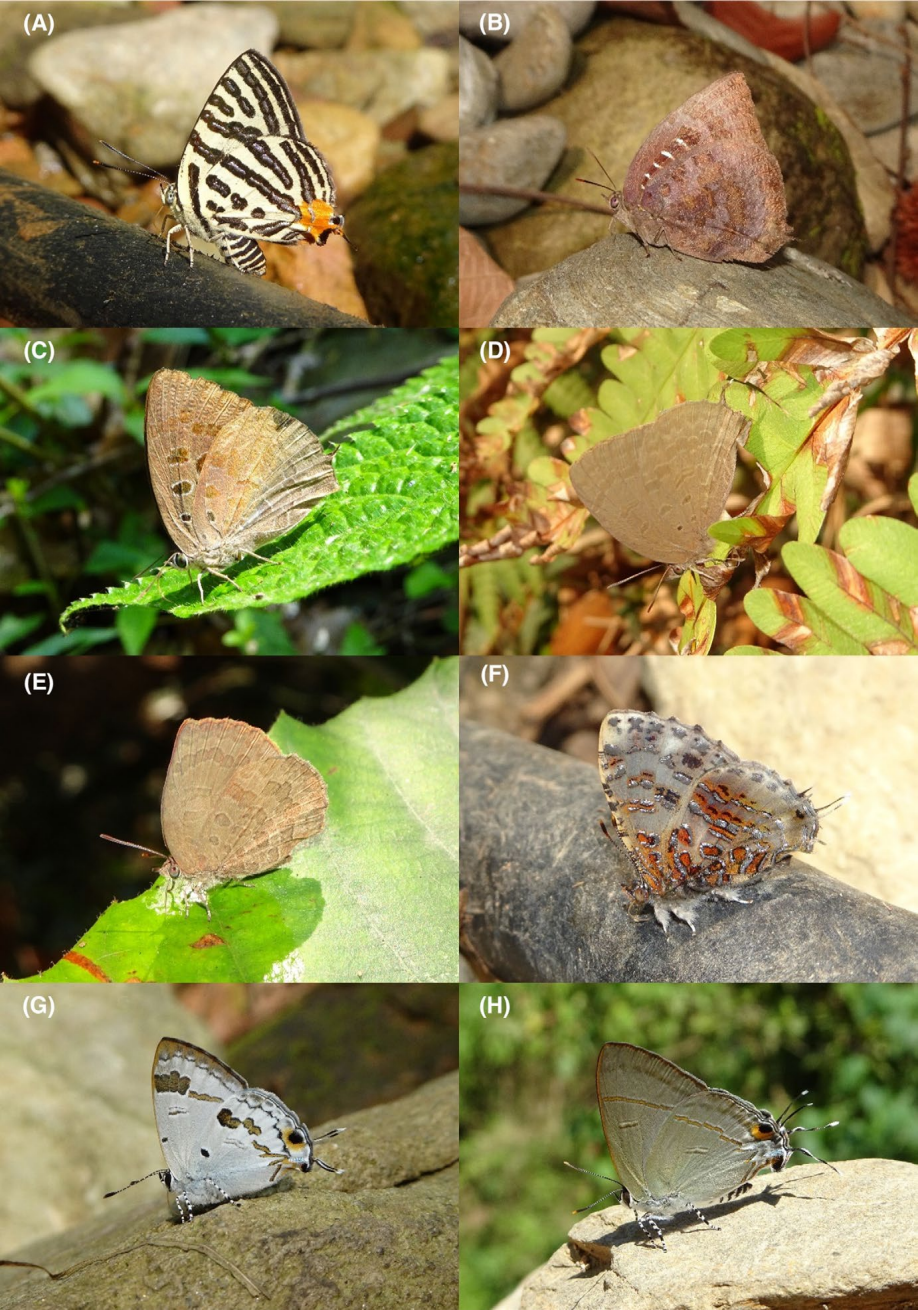
Lycaenidae of Bhorletar. (A) *Spindasis syama peguanus* Moore, 1884 – Club Silverline; (B) *Arhopala centaurus pirithous* (Moore, [1884])—Centaur Oakblue; (C) *Arhopala eumolphus eumolphus* (Cramer, [1780])—Green Oakblue; (D) *Arhopala oenea* (Hewitson, 1869)—Hewitson's Dull Oakblue; (E) *Arhopala paramuta paramuta* (de Nicéville, [1884])—Hooked Oakblue; (F) *Catapaecilma major major* Druce, 1895—Common Tinsel; (G) *Chliaria othona othona* (Hewitson, 1865)—Orchid Tit; (H) *Hypolycaena erylus himavantus* Fruhstorfer, 1912—Common Tit.

**FIGURE 14:**
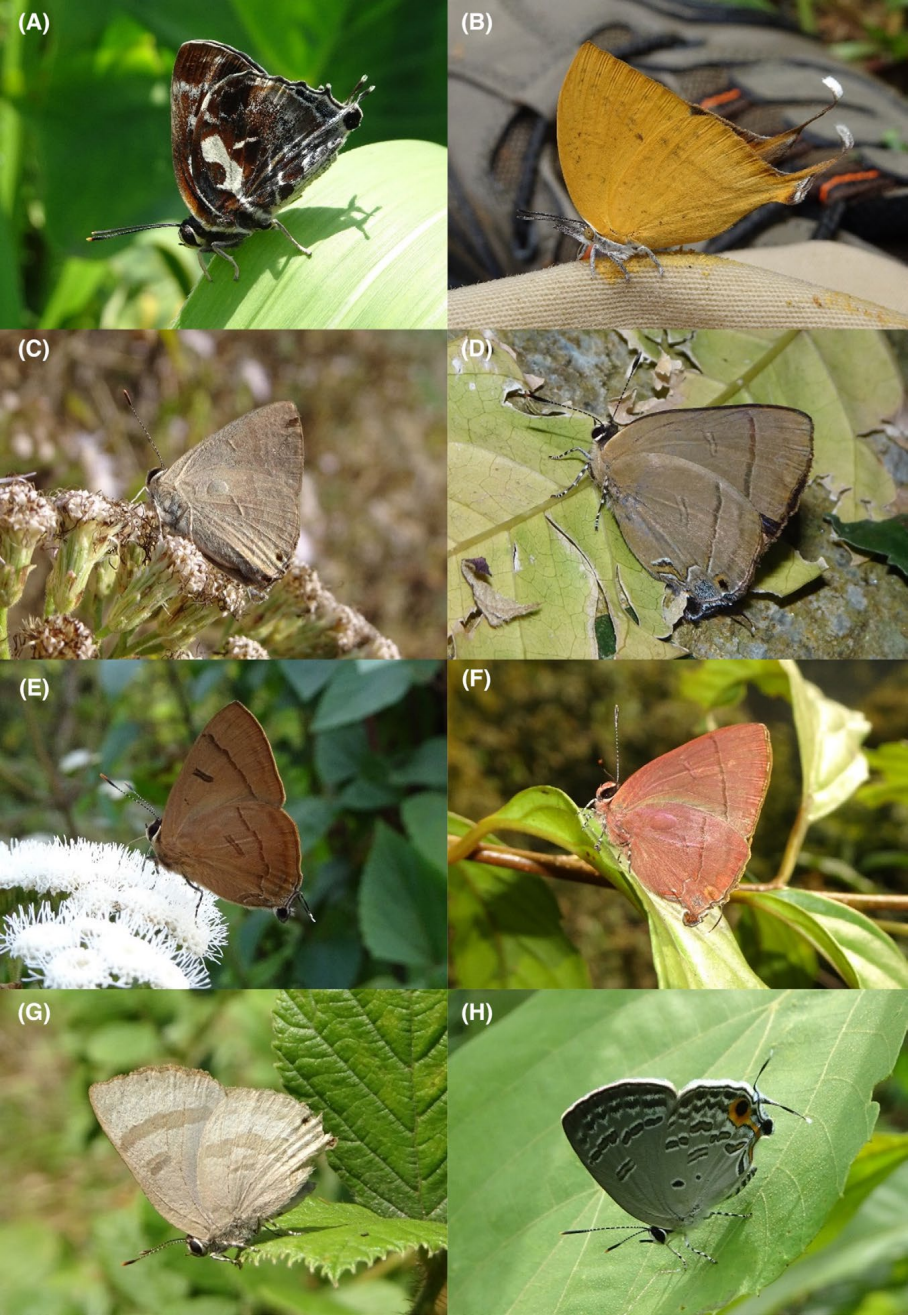
Lycaenidae of Bhorletar. (A) *Iraota timoleon timoleon* (Stoll, [1790])—Silverstreak Blue; (B) *Loxura atymnus atymnus* (Stoll, 1780)—Yamfly; (C) *Rapala manea schistacea* (Moore, 1879)—Slate Flash; (D) *Rapala nissa nissa* (Kollar, [1844])—Common Flash; (E) *Rapala pheretima petosiris* (Hewitson, 1863)—Copper Flash; (F) *Rapala* cf. *rectivitta* (Moore, 1879)—Shot Flash; (G) *Rapala varuna gebenia* Fruhstorfer, 1914—Indigo Flash; (H) *Sinthusa chandrana chandrana* (Moore, 1882)—Broad Spark.

**FIGURE 15:**
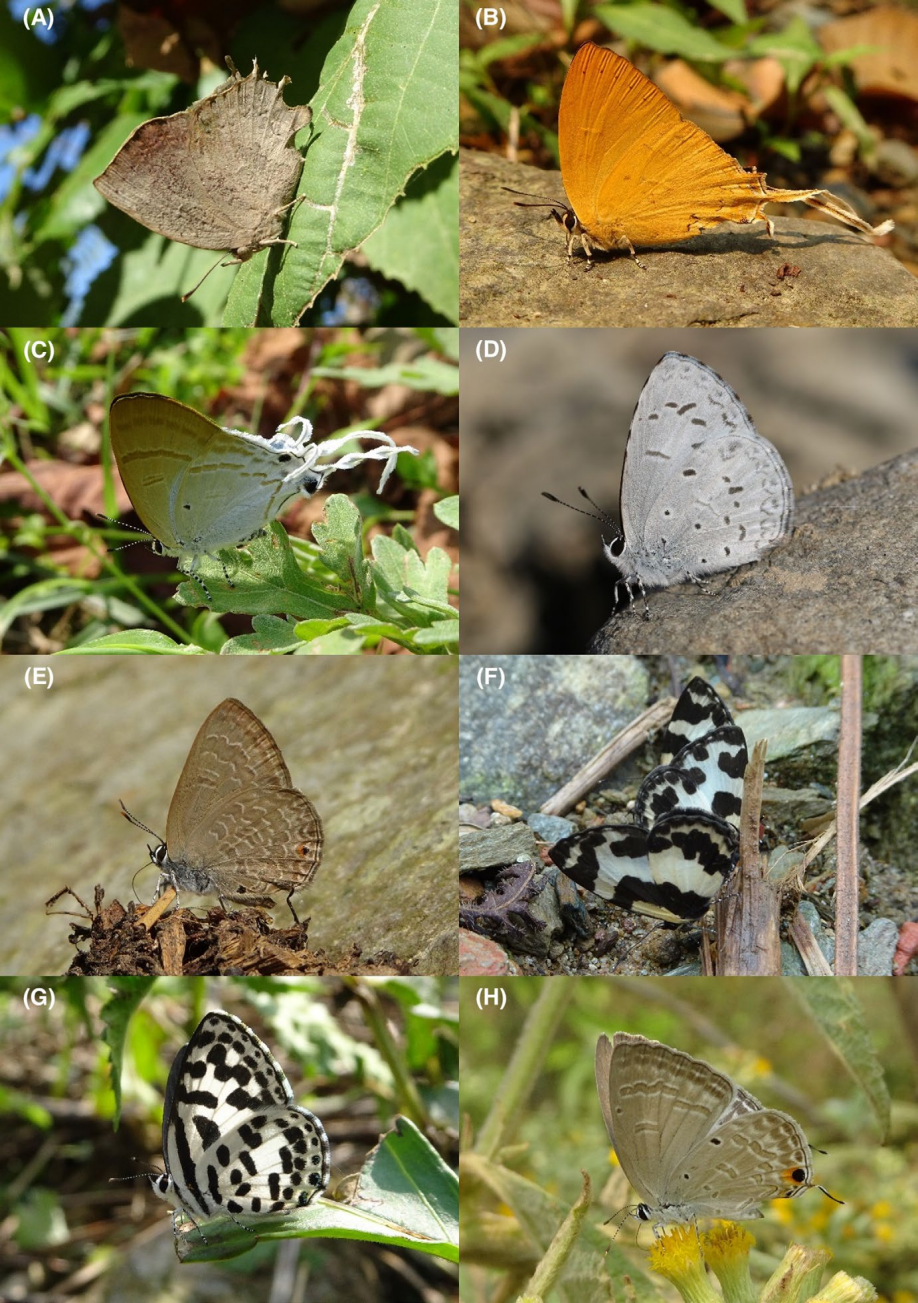
Lycaenidae of Bhorletar. (A) *Surendra quercetorum quercetorum* (Moore, [1858])—Common Acacia Blue; (B) *Ticherra acte acte* (Moore, [1858])—Blue Imperial; (C) *Zeltus amasa amasa* (Hewitson, 1865)—Fluffy Tit; (D) *Acytolepis puspa gisca* (Fruhstorfer, 1910)—Common Hedge Blue; (E) *Anthene emolus emolus* (Godart, [1824])—Ciliate Blue; (F) *Caleta elna noliteia* (Fruhstorfer, 1918)—Elbowed Pierrot; (G) *Castalius rosimon rosimon* (Fabricius, 1775)—Common Pierrot; (H) *Catochrysops strabo strabo* (Fabricius, 1793)—Forget‐Me‐Not‐Blue.

**FIGURE 16:**
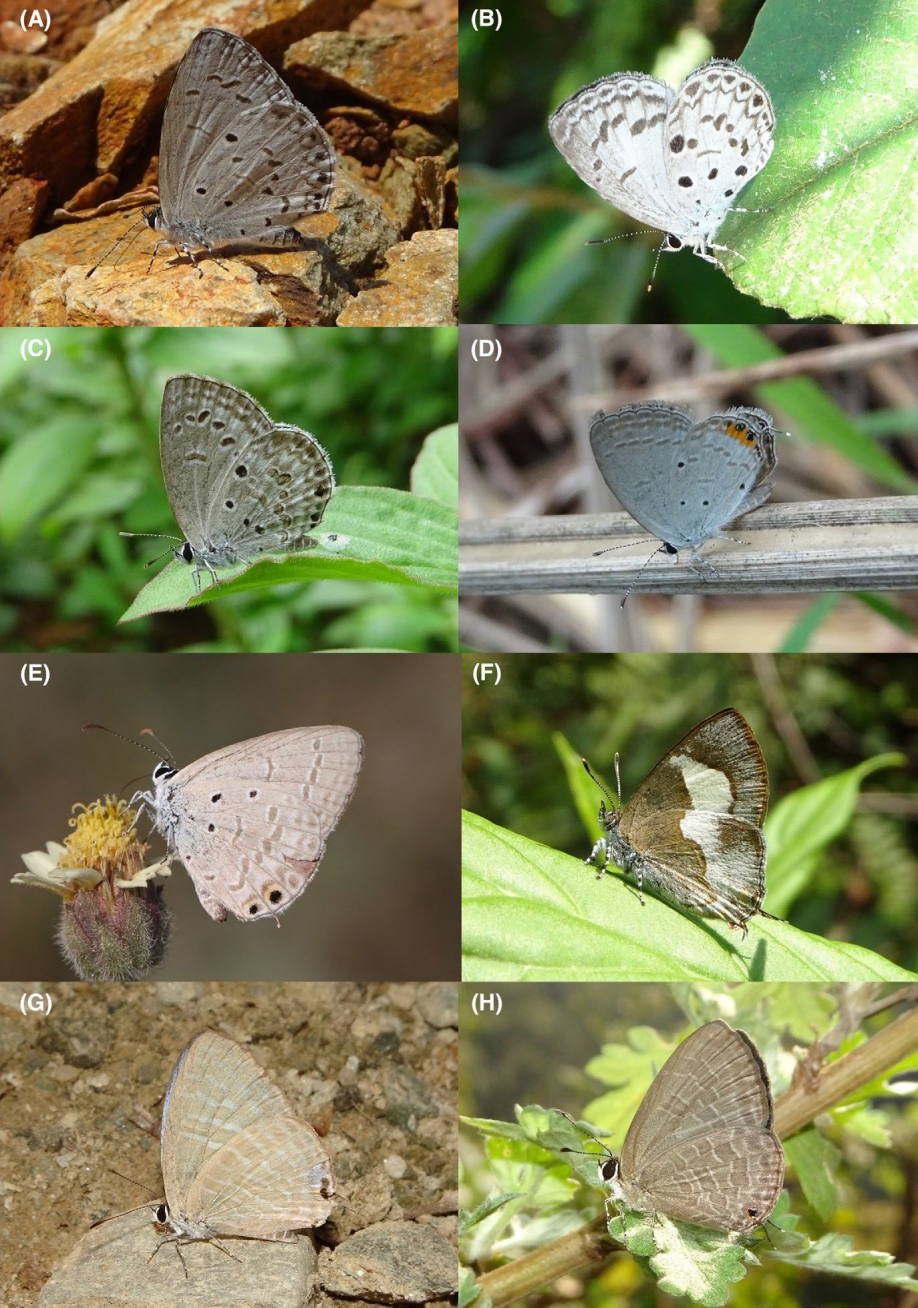
Lycaenidae of Bhorletar. (A) *Celastrina lavendularis limbata* (Moore, 1879)—Plain Hedge Blue; (B) *Celatoxia marginata* (de Nicéville, 1884) Margined Hedge Blue; (C) *Chilades lajus lajus* (Stoll, [1780])—Lime Blue; (D) *Cupido lacturnus assamica* Tytler, 1915—Indian Cupid; (E) *Euchrysops cnejus cnejus* (Fabricius, 1798)—Gram Blue; (F) *Horaga onyx onyx* (Moore, 1858)—Common Onyx; (G) *Jamides alecto alocina* Swinhoe, 1915—Metallic Cerulean; (H) *Jamides bochus bochus* (Stoll, [1782])—Dark Cerulean.

**FIGURE 17:**
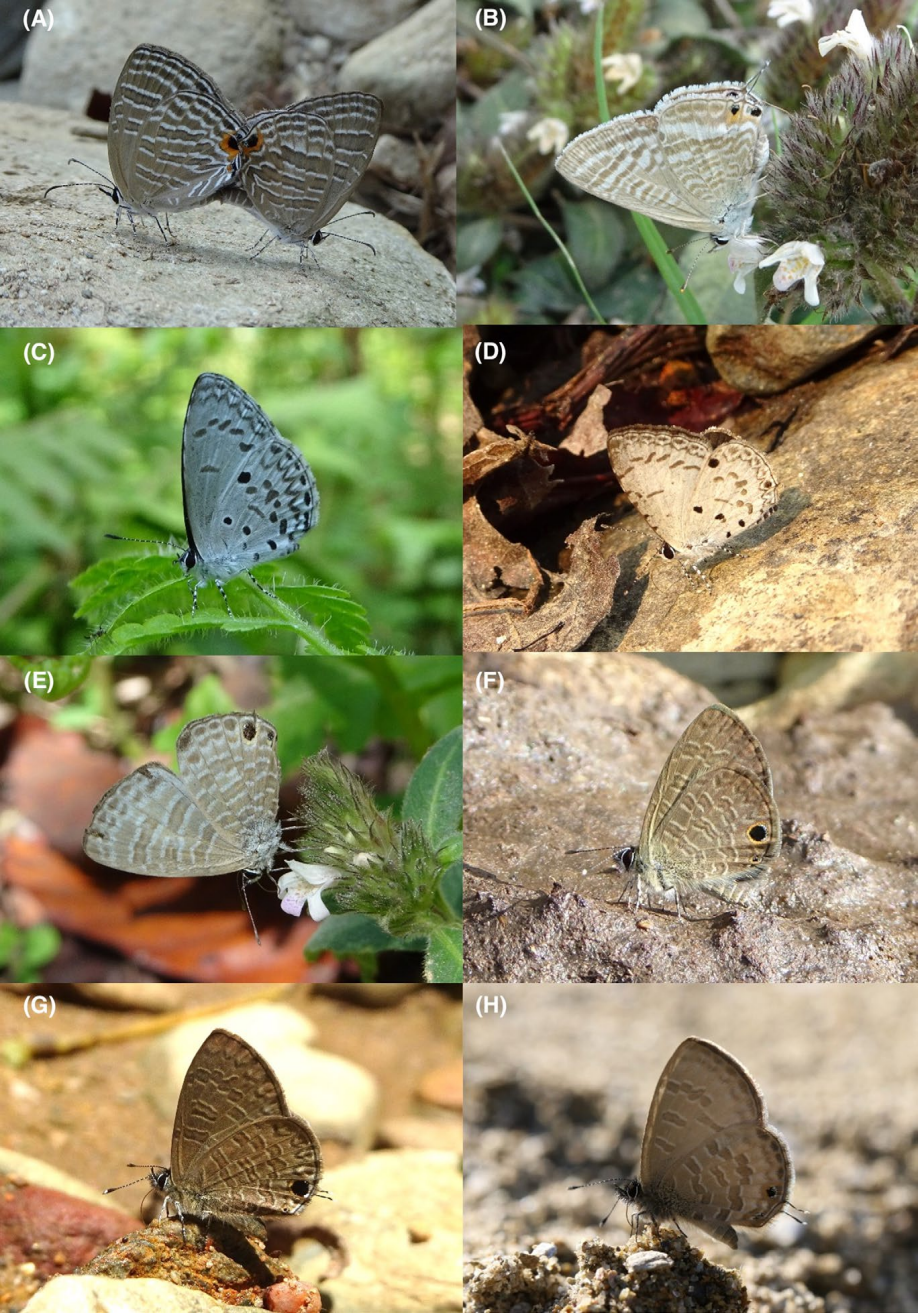
Lycaenidae of Bhorletar. (A) *Jamides celeno aelianus* (Fabricius, 1793)—Common Cerulean; (B) *Lampides boeticus* (Linnaeus, 1767)—Pea Blue; (C) *Lestranicus transpectus* (Moore, 1879)—White‐Banded Hedge Blue; (D) *Megisba malaya sikkima* Moore, 1884—Malayan; (E) *Nacaduba kurava euplea* Fruhstorfer, 1916—Transparent Sixline‐Blue; (F) *Prosotas dubiosa indica* (Evans, [1925])—Tailless Lineblue; (G) *Prosotas nora nora* (C. Felder, 1860)—Common Lineblue; (H) *Prosotas pia marginata* Tite, 1963—Margined Lineblue.

**FIGURE 18:**
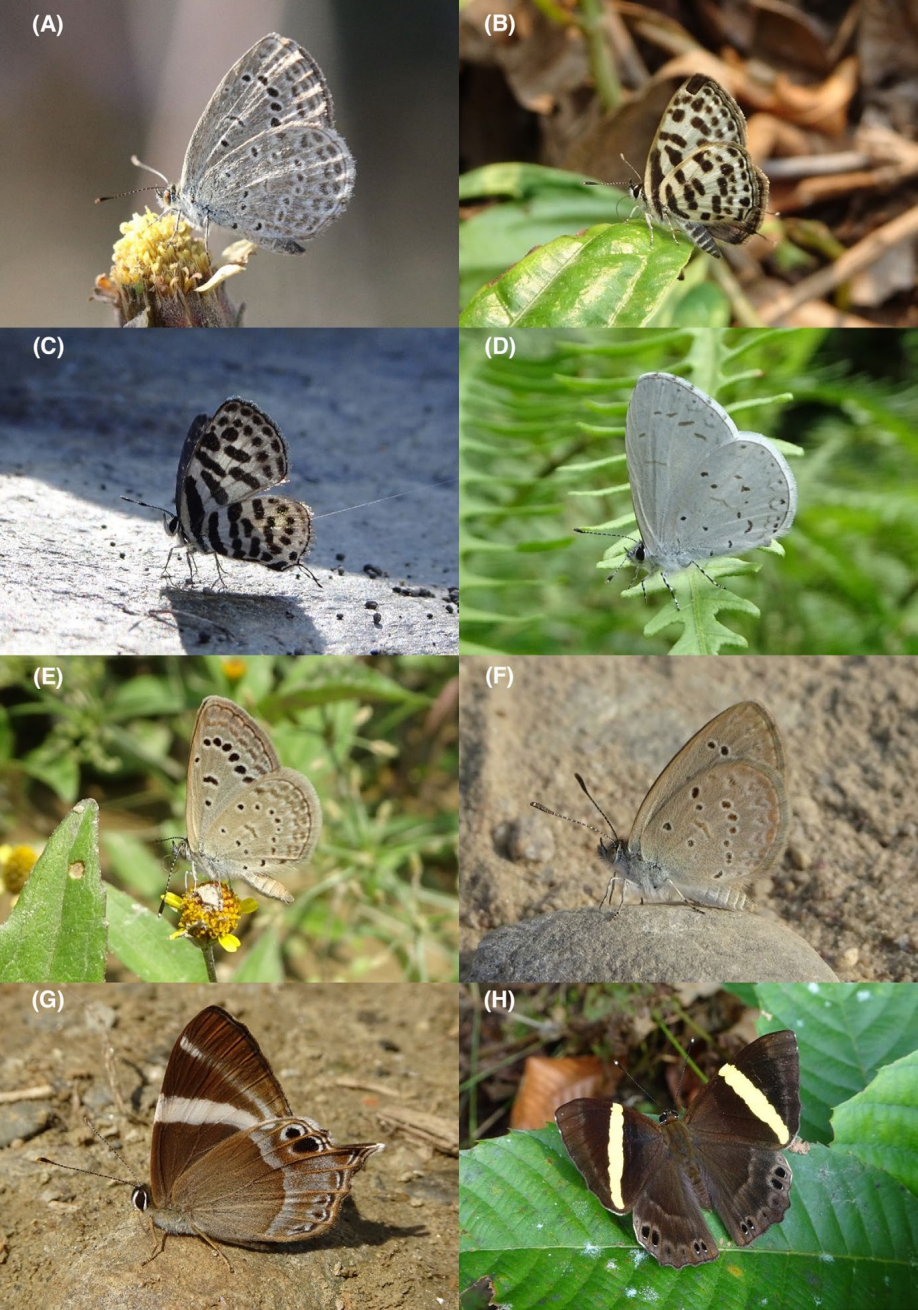
Lycaenidae and Riodinidae of Bhorletar. (A) *Pseudozizeeria maha maha* (Kollar, [1844])—Pale Grass Blue; (B) *Tarucus ananda* (de Nicéville, [1884])—Dark Pierrot; (C) *Tarucus waterstradti dharta* Bethune‐Baker, [1918]—Assam Pierrot; (D) *Udara dilecta dilecta* (Moore, 1879)—Pale Hedge Blue; (E) *Zizeeria karsandra* (Moore, 1865)—Dark Grass Blue; (F) *Zizina otis indica* (Murray, 1874)—Lesser Grass Blue; (G) *Abisara chela chela* de Nicéville, 1886—Spot Judy; (H) *Abisara fylla* (Westwood, 1851)—Dark Judy.

**FIGURE 19:**
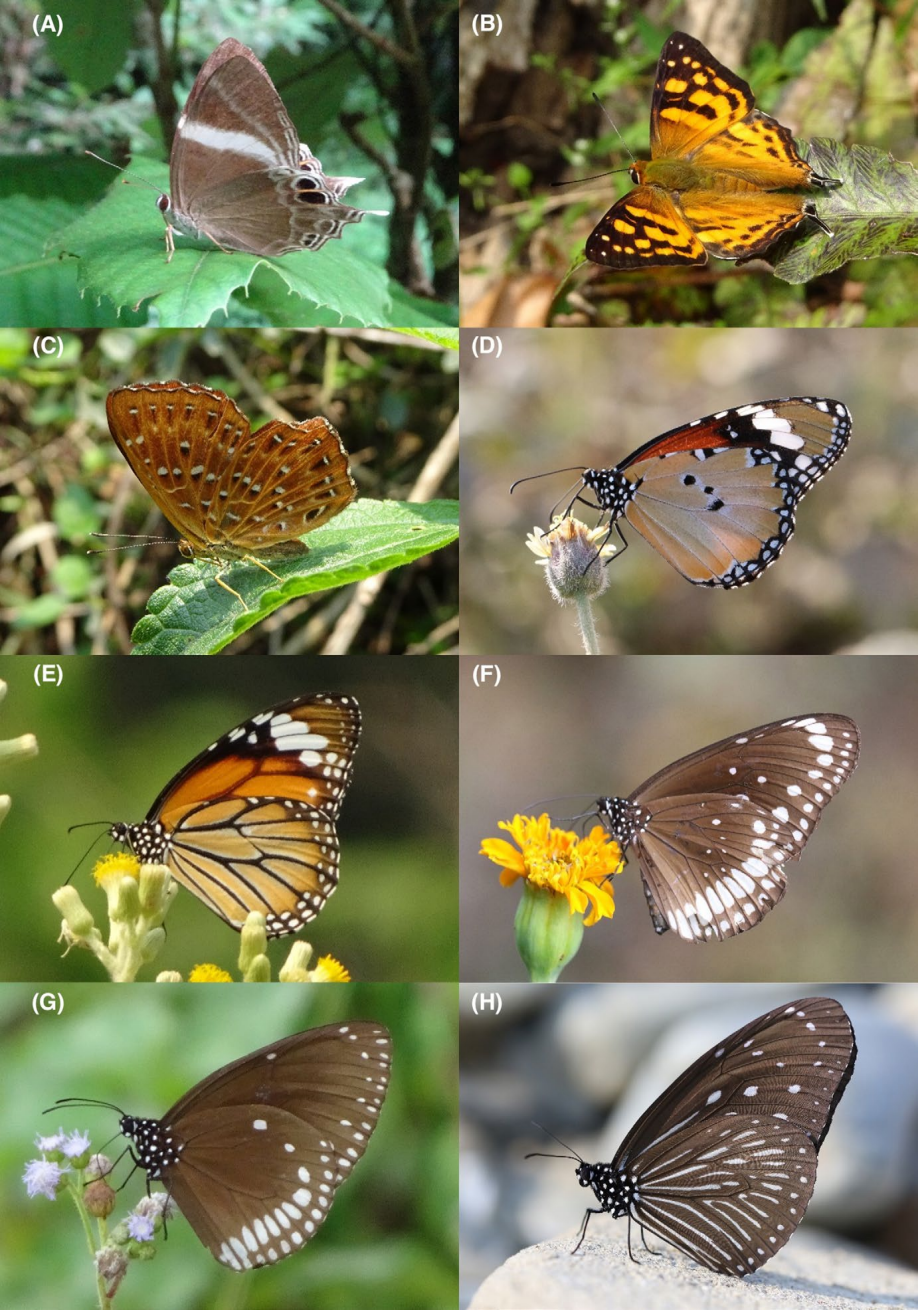
Riodinidae and Nymphalidae of Bhorletar. (A) *Abisara neophron neophronides* Fruhstorfer, 1914—Tailed Judy; (B) *Dodona egeon egeon* (Westwood, [1851])—Orange Punch; (C) *Zemeros flegyas indicus* Fruhstorfer, 1898—Punchinello; (D) *Danaus chrysippus chrysippus* (Linnaeus, 1758)—Plain Tiger; (E) *Danaus genutia genutia* (Cramer, [1779])—Common Tiger; (F) *Euploea core core* (Cramer, [1780])—Common Crow; (G) *Euploea klugii kollari* C. & R. Felder, [1865]—Brown King Crow; (H) *Euploea mulciber mulciber* (Cramer, [1777])—Striped Blue Crow.

**FIGURE 20:**
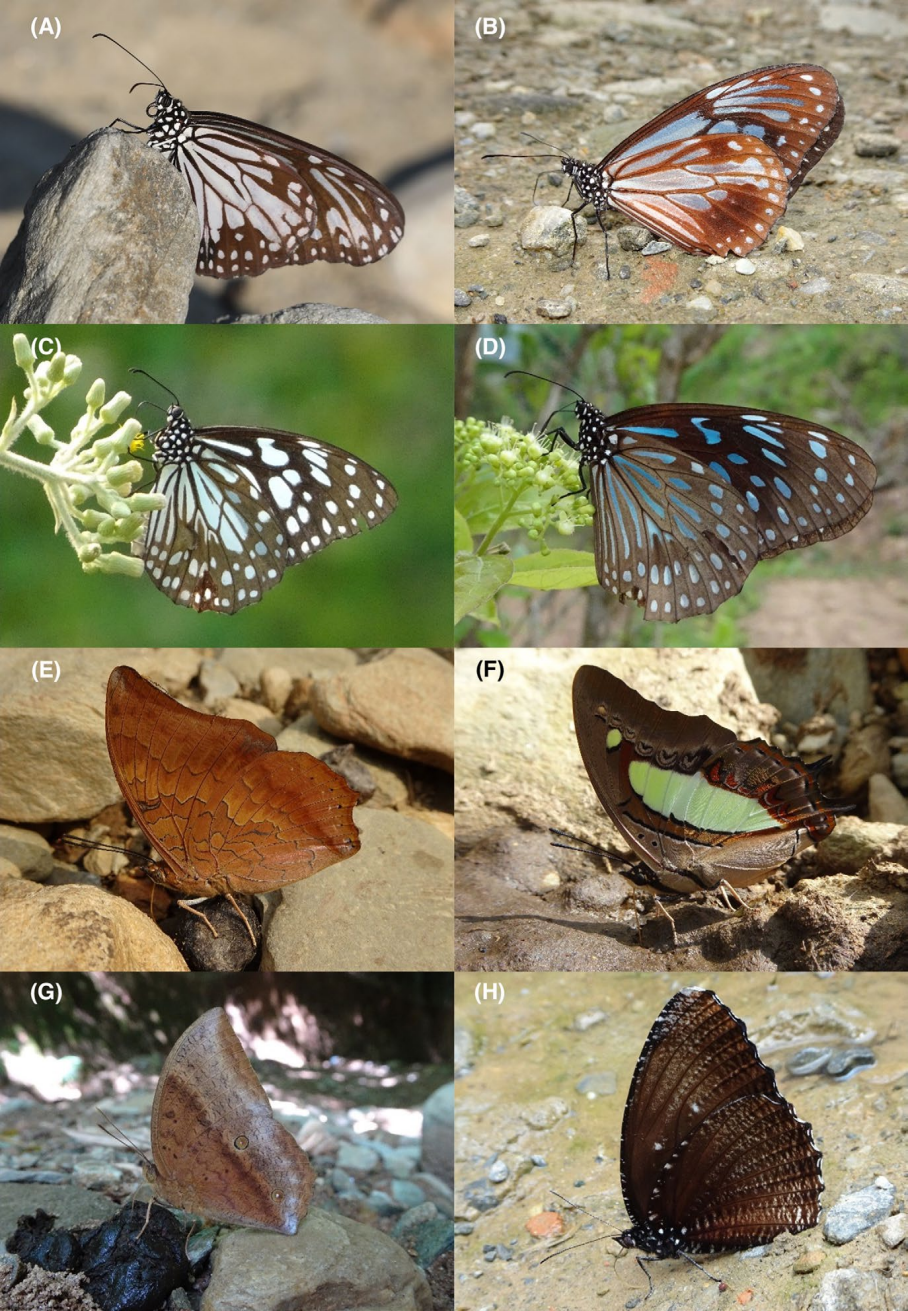
Nymphalidae of Bhorletar. (A) *Parantica aglea melanoides* Moore, 1883—Glassy Tiger; (B) *Parantica melaneus plataniston* (Fruhstorfer, 1910)—Chocolate Tiger; (C) *Tirumala limniace exoticus* (Gmelin, 1790)—Blue Tiger; (D) *Tirumala septentrionis septentrionis* (Butler, 1874)—Dark Blue Tiger; (E) *Charaxes bernardus hierax* C. & R. Felder, [1867]—Tawny Rajah; (F) *Polyura athamas athamas* (Drury, [1773])—Common Nawab; (G) *Discophora sondaica zal* Westwood, 1851—Common Duffer; (H) *Elymnias malelas malelas* (Hewitson, 1863)—Spotted Palmfly.

**FIGURE 21:**
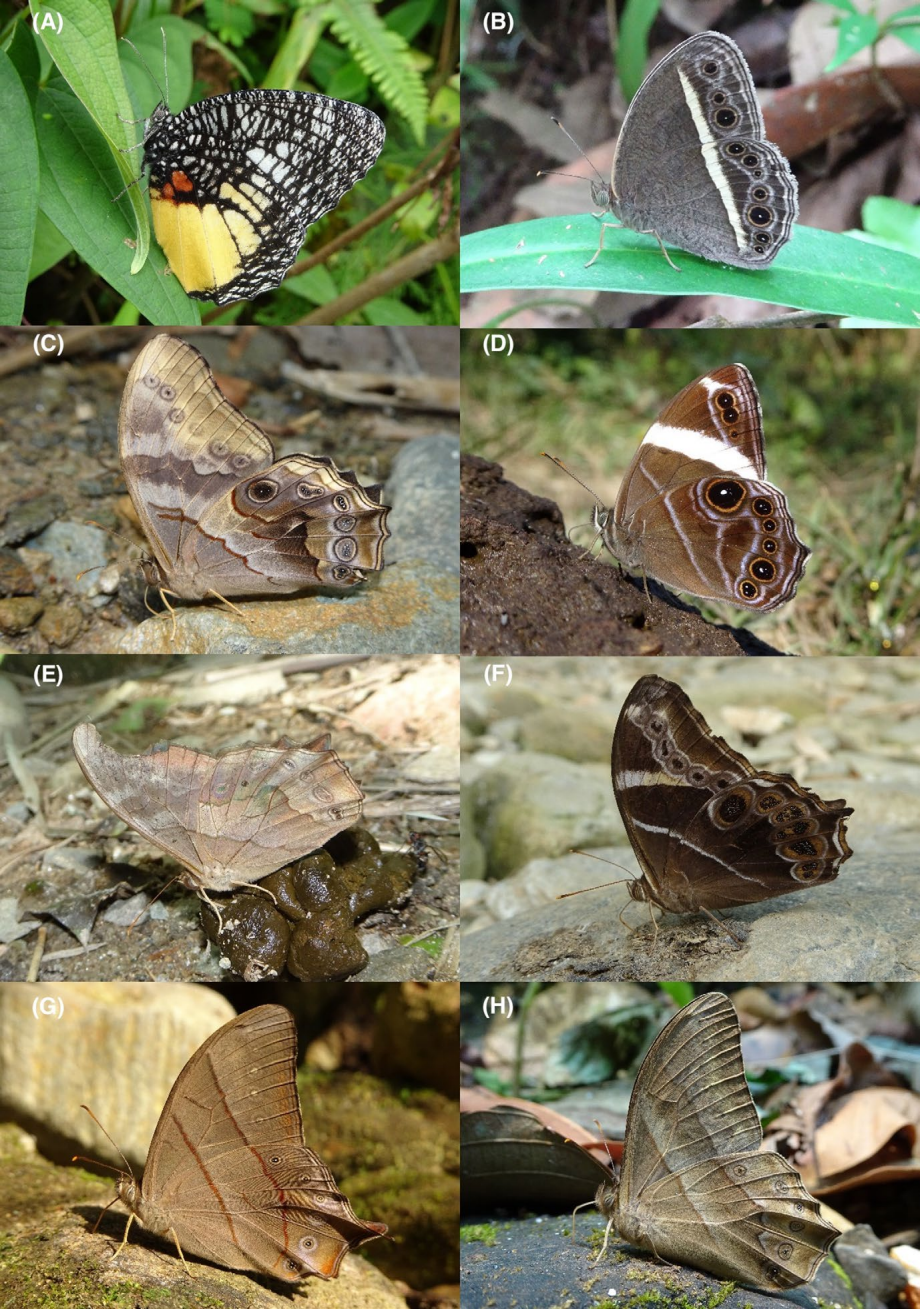
Nymphalidae of Bhorletar. (A) *Elymnias vasudeva vasudeva* Moore, 1857—Jezebel Palmfly; (B) *Heteropsis malsara* (Moore, 1857)—White‐Line Bushbrown; (C) *Lethe chandica chandica* (Moore, [1858])—Angled Red Forester; (D) *Lethe confusa confusa* Aurivillius, 1898—Banded Treebrown; (E) *Lethe distans* Butler, 1870—Scarce Red Forester; (F) *Lethe europa niladana* Fruhstorfer, 1911—Bamboo Treebrown; (G) *Lethe kansa kansa* (Moore, 1857)—Bamboo Forester; (H) *Lethe mekara mekara* (Moore, [1858])—Common Red Forester.

**FIGURE 22:**
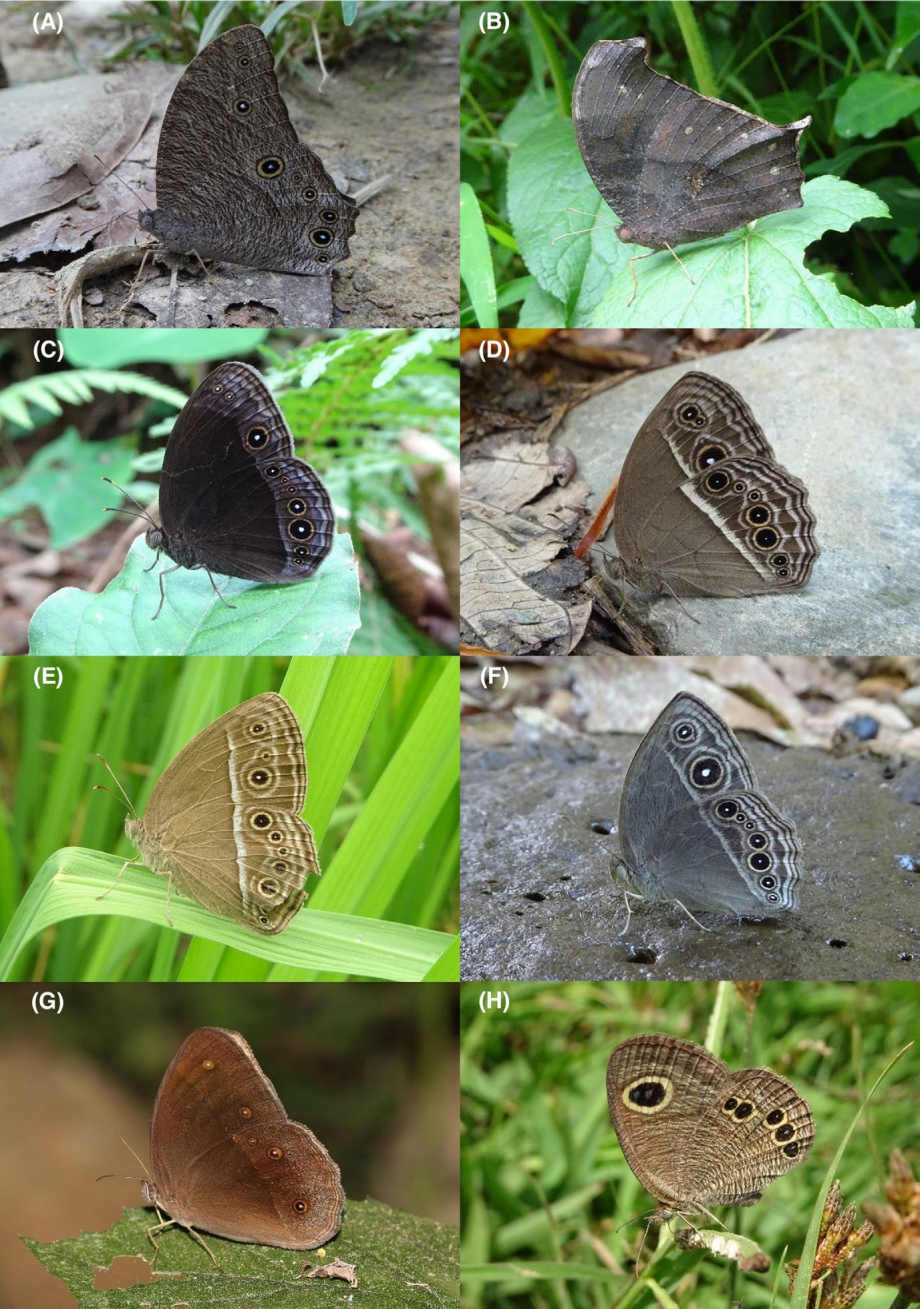
Nymphalidae of Bhorletar. (A) *Melanitis leda leda* (Linnaeus, 1758)—Common Evening Brown; (B) *Melanitis phedima bela* Moore, 1857—Dark Evening Brown; (C) *Mycalesis adamsoni adamsoni* Watson, 1897—Watson's Bushbrown; (D) *Mycalesis mineus mineus* (Linnaeus, 1758)—Dark‐Brand Bushbrown; (E) *Mycalesis perseus blasius* (Fabricius, 1798)—Common Bushbrown; (F) *Mycalesis visala visala* Moore, [1858]—Long‐Brand Bushbrown; (G) *Orsotriaena medus medus* (Fabricius, 1775)—Jungle Brown; (H) *Ypthima avanta* Moore, [1875]—Jewel Fivering.

**FIGURE 23:**
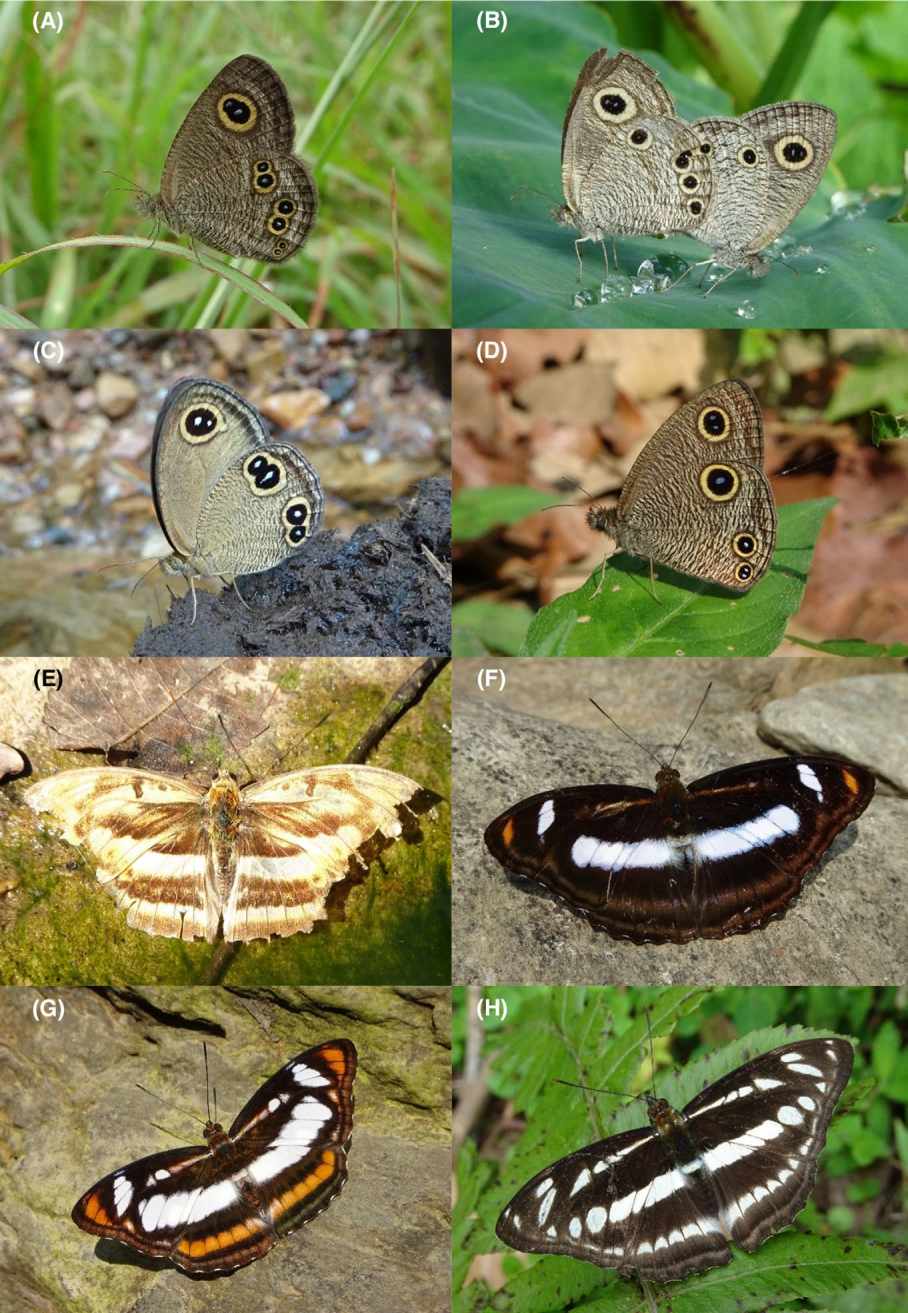
Nymphalidae of Bhorletar. (A) *Ypthima baldus baldus* (Fabricius, 1775)—Common Fivering; (B) *Ypthima huebneri* Kirby, 1871—Common Fourring; (C) *Ypthima hyagriva nepalica* Smith, 1983—Brown Argus; (D) *Ypthima newara newara* Moore, [1875]—Newar Threering; (E) *Abrota ganga ganga* Moore, 1857—Sergeant Major; (F) *Athyma cama cama* Moore, [1858]—Orange Staff Sergeant; (G) *Athyma nefte inara* (Westwood, 1850)—Color Sergeant; (H) *Athyma orientalis* Elwes, 1888—Oriental Sergeant.

**FIGURE 24:**
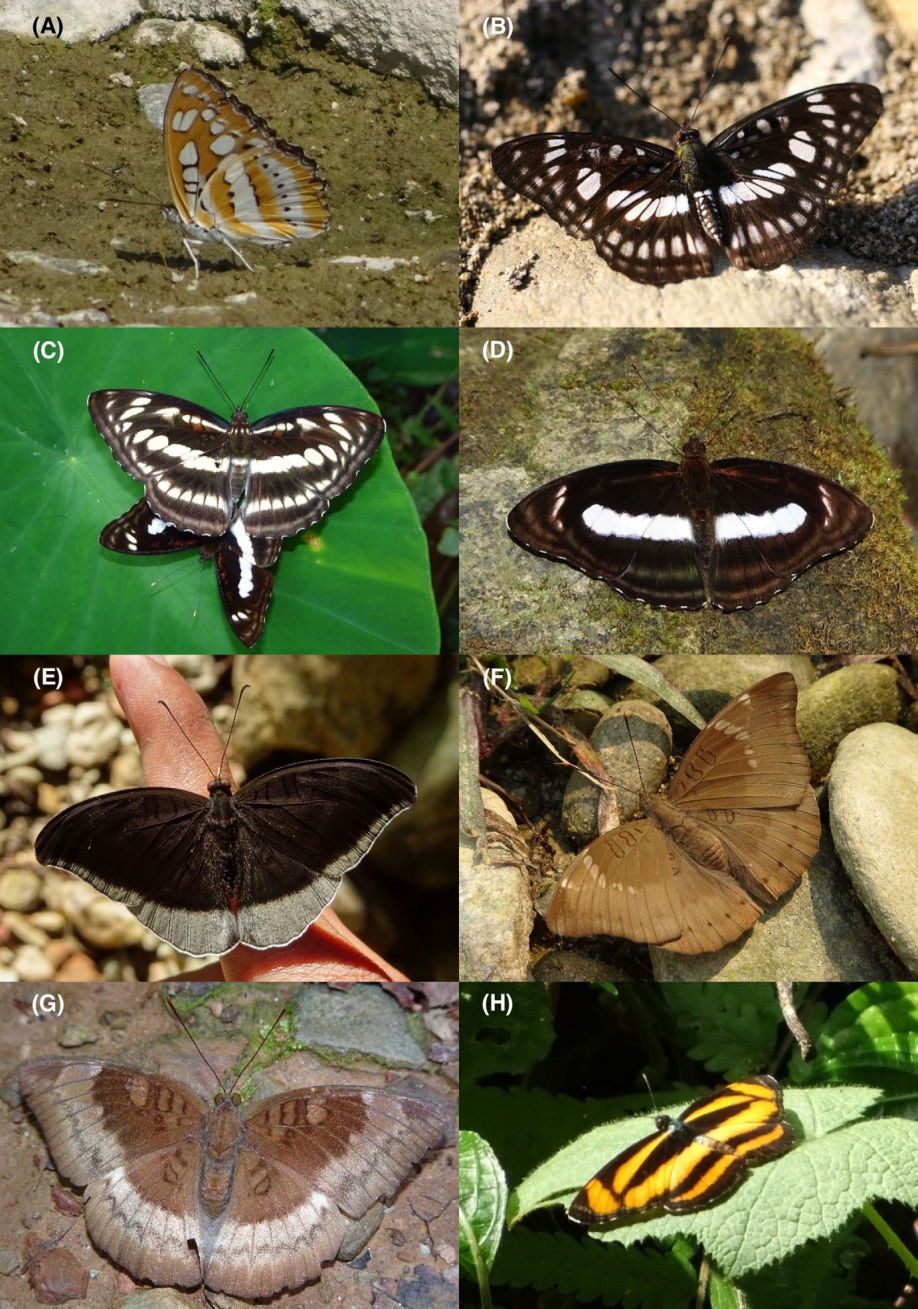
Nymphalidae of Bhorletar. (A) *Athyma perius perius* (Linnaeus, 1758)—Common Sergeant; (B) *Athyma ranga ranga* Moore, [1858]—Blackvein Sergeant; (C) *Athyma selenophora selenophora* (Kollar, [1844])—Staff Sergeant; (D) *Athyma zeroca zeroca* Moore, 1872—Small Staff Sergeant; (E) *Cynitia lepidea lepidea* (Butler, 1868)—Grey Count; (F) *Euthalia aconthea suddhodana* Fruhstorfer, 1913—Common Baron; (G) *Euthalia monina kesava* (Moore, 1859)—Powdered Baron; (H) *Lasippa viraja viraja* (Moore, 1872)—Yellowjack Sailer.

**FIGURE 25:**
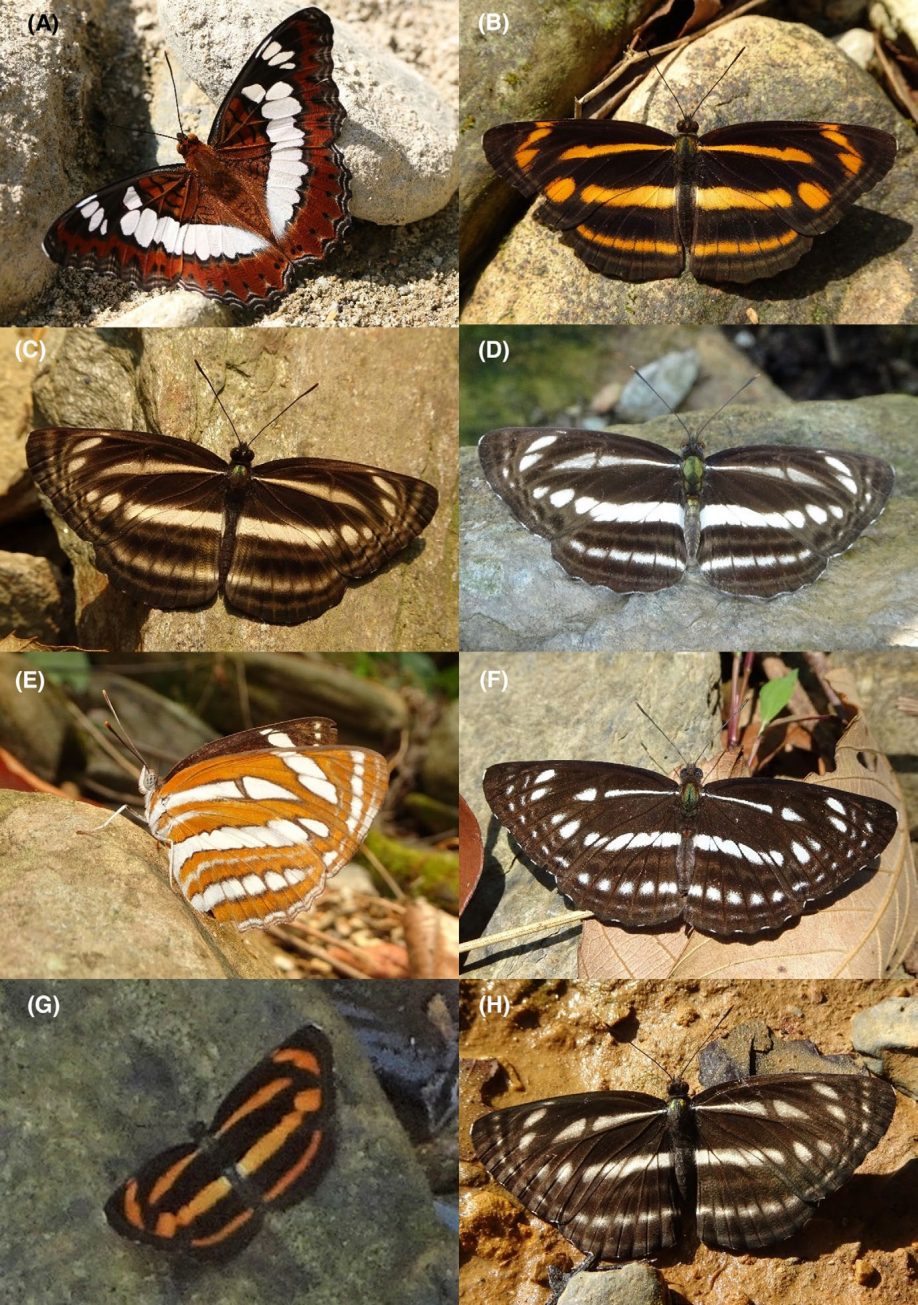
Nymphalidae of Bhorletar. (A) *Moduza procris procris* (Cramer, [1777])—Commander; (B) *Neptis ananta ochracea* Evans, 1924—Yellow Sailer; (C) *Neptis cartica cartica* Moore, 1872—Plain Sailer; (D) *Neptis clinia susruta* Moore, 1872—Sullied Sailer; (E) *Neptis hylas kamarupa* Moore, [1875]—Common Sailer; (F) *Neptis magadha khasiana* Moore, 1872—Spotted Sailer; (G) *Neptis miah miah* Moore, 1857—Small Yellow Sailer; (H) *Neptis nata adipala* Moore, 1872—Clear Sailer.

**FIGURE 26:**
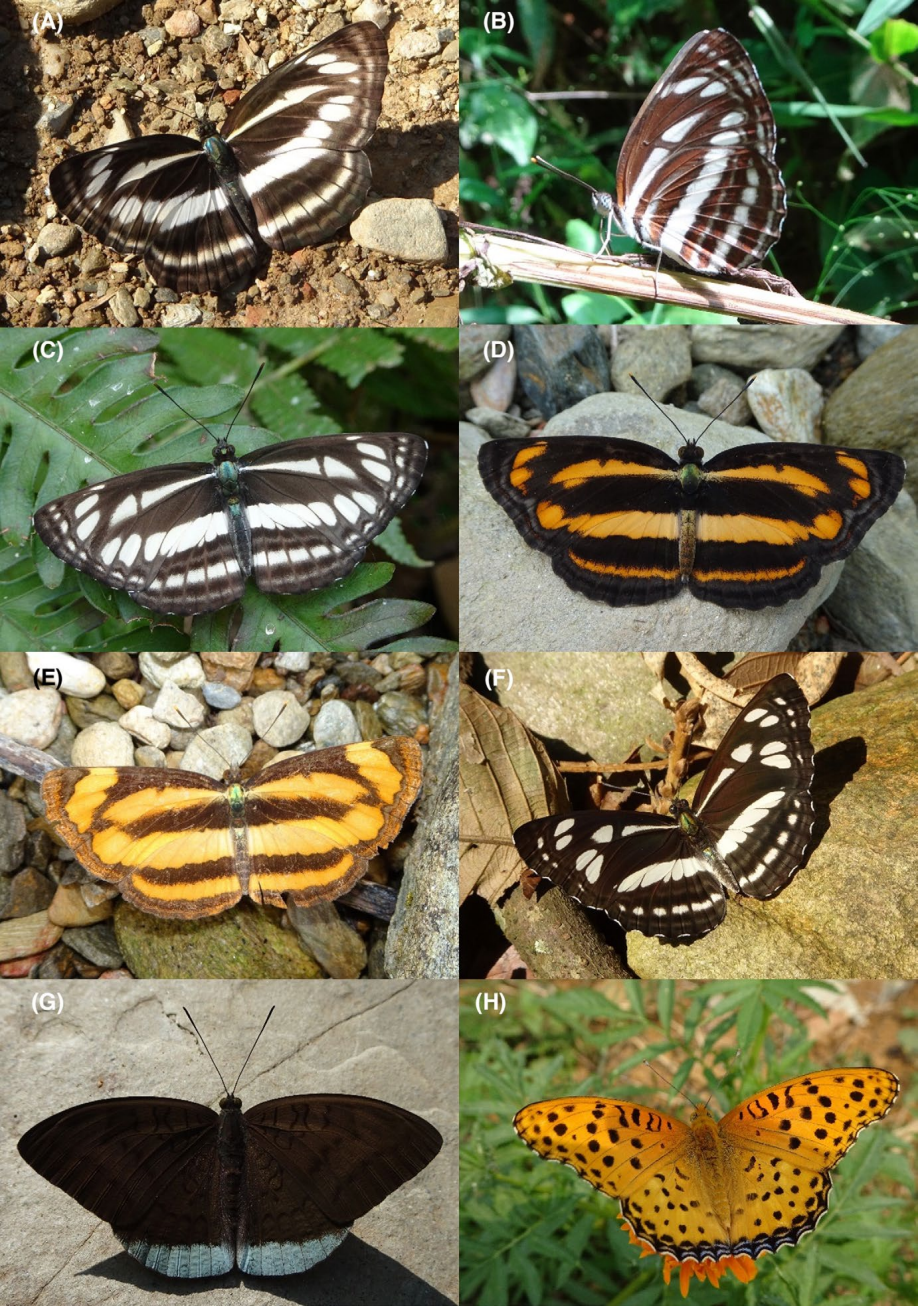
Nymphalidae of Bhorletar. (A) *Neptis sankara amba* Moore, 1858—Broad‐Banded Sailer; (B) *Neptis sappho astola* Moore, 1872—Pallas' Sailer; (C) *Neptis soma butleri* Eliot, 1969—Creamy Sailer; (D) *Pantoporia hordonia hordonia* (Stoll, [1790])—Common Lascar; (E) *Pantoporia sandaka davidsoni* Eliot, 1969—Extra Lascar; (F) *Phaedyma columella ophiana* (Moore, 1872)—Short‐Banded Sailer; (G) *Tanaecia julii appiades* (Ménétriés, 1857)—Common Earl; (H) *Argynnis hyperbius hyperbius* (Linnaeus, 1763)—Indian Fritillary.

**FIGURE 27:**
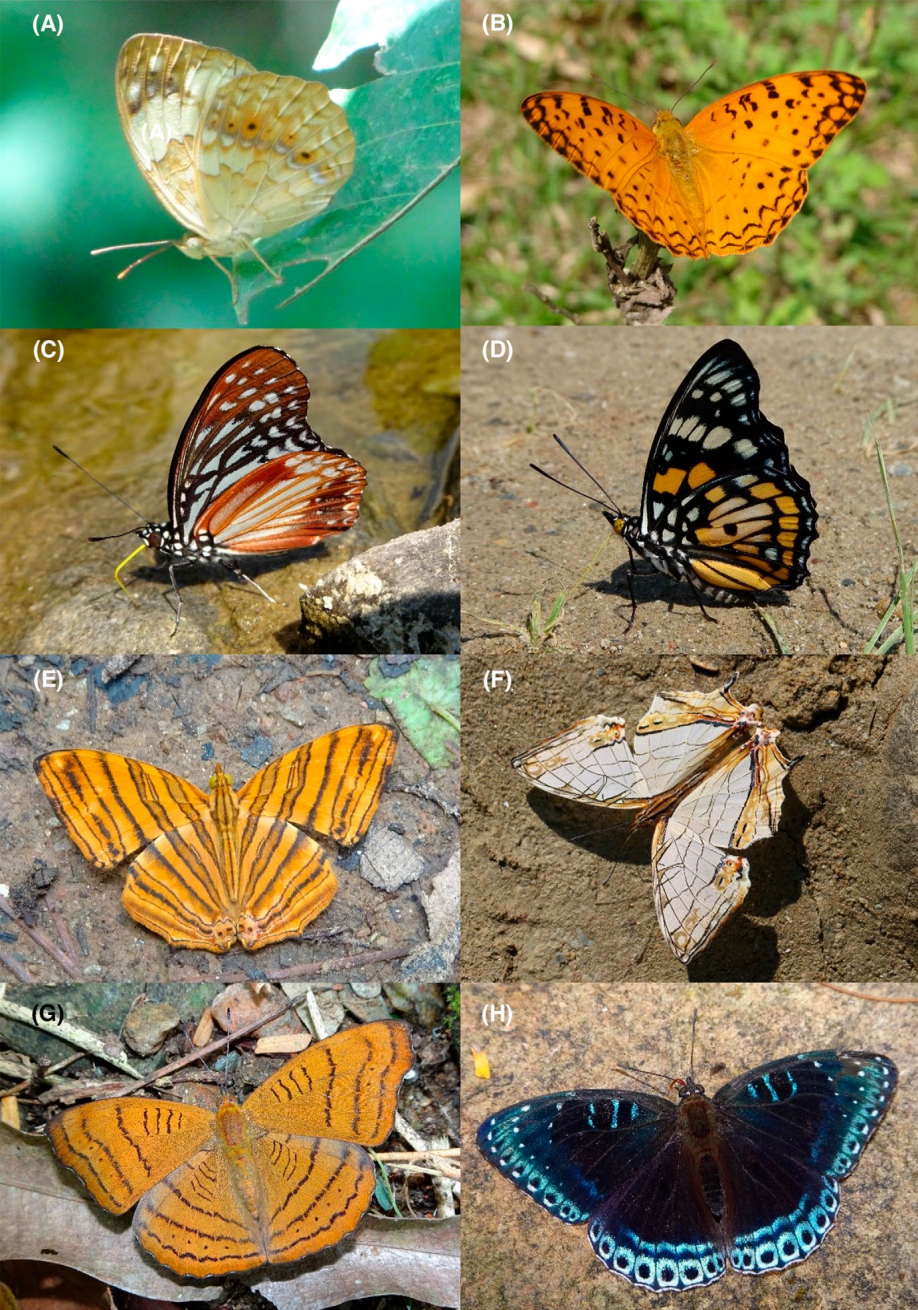
Nymphalidae of Bhorletar. (A) *Cupha erymanthis lotis* (Sulzer, 1776)—Rustic; (B) *Phalanta phalantha phalantha* (Drury, [1773])—Common Leopard; (C) *Hestinalis nama nama* (Doubleday, 1844)—Circe; (D) *Sephisa chandra chandra* (Moore, [1858])—Eastern Courtier; (E) *Chersonesia risa risa* (Doubleday, [1848])—Common Maplet; (F) *Cyrestis thyodamas thyodamas* Boisduval, 1846—Common Map; (G) *Pseudergolis wedah wedah* (Kollar, 1848)—Tabby; (H) *Stibochiona nicea nicea* (Gray, 1846)—Popinjay.

**FIGURE 28:**
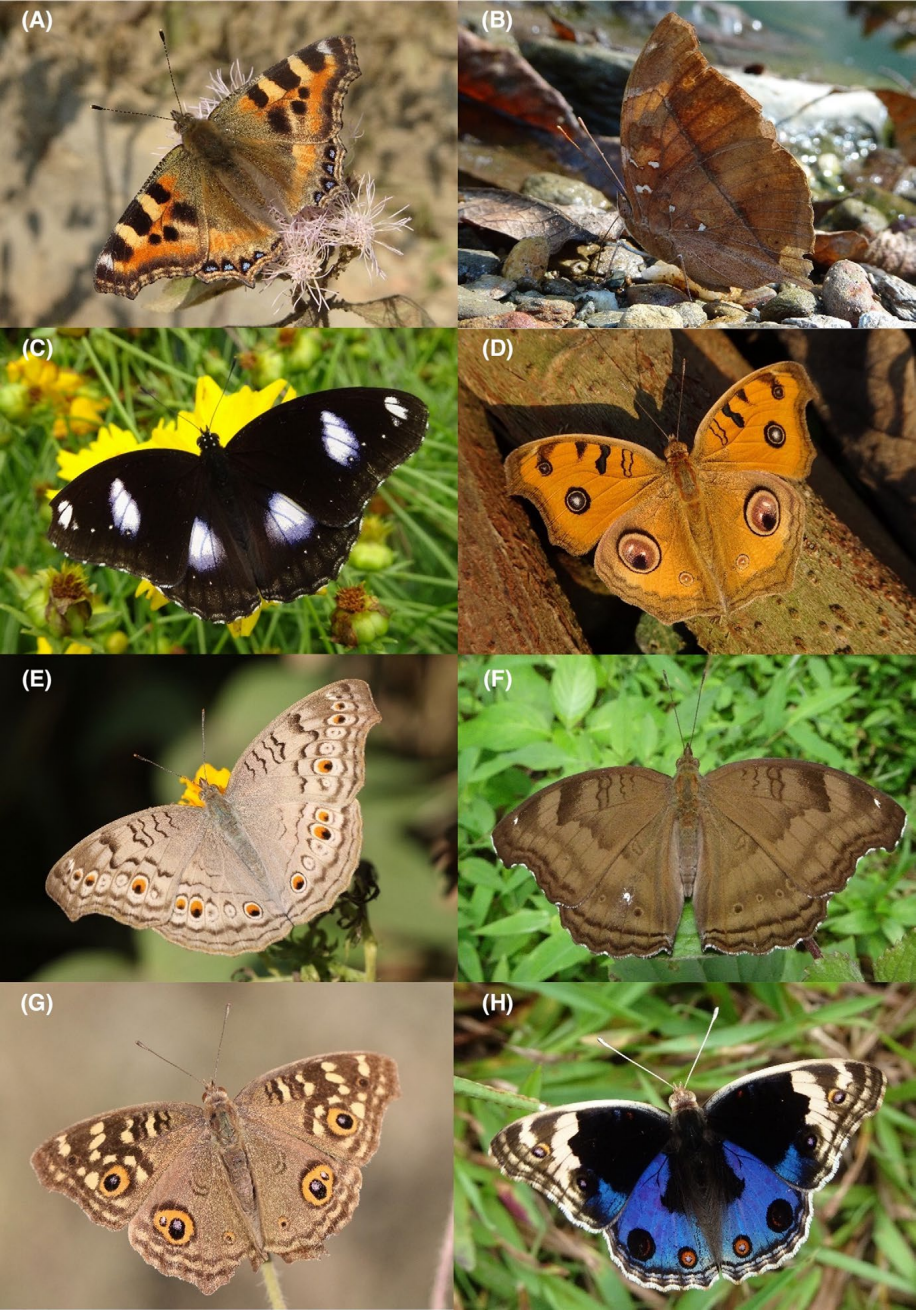
Nymphalidae of Bhorletar. (A) *Aglais caschmirensis aesis* Fruhstorfer, 1912—Indian Tortoiseshell; (B) *Doleschallia bisaltide* (Cramer, [1777])—Autumn Leaf; (C) *Hypolimnas bolina jacintha* (Drury, 1773)—Great Eggfly; (D) *Junonia almana almana* (Linnaeus, 1758)—Peacock Pansy; (E) *Junonia atlites atlites* (Linnaeus, 1763)—Grey Pansy; (F) *Junonia iphita iphita* (Cramer, [1779])—Chocolate Pansy; (G) *Junonia lemonias persicaria* (Fruhstorfer, 1912)—Lemon Pansy; (H) *Junonia orithya ocyale* Hübner, [1819]—Blue Pansy.

**FIGURE 29:**
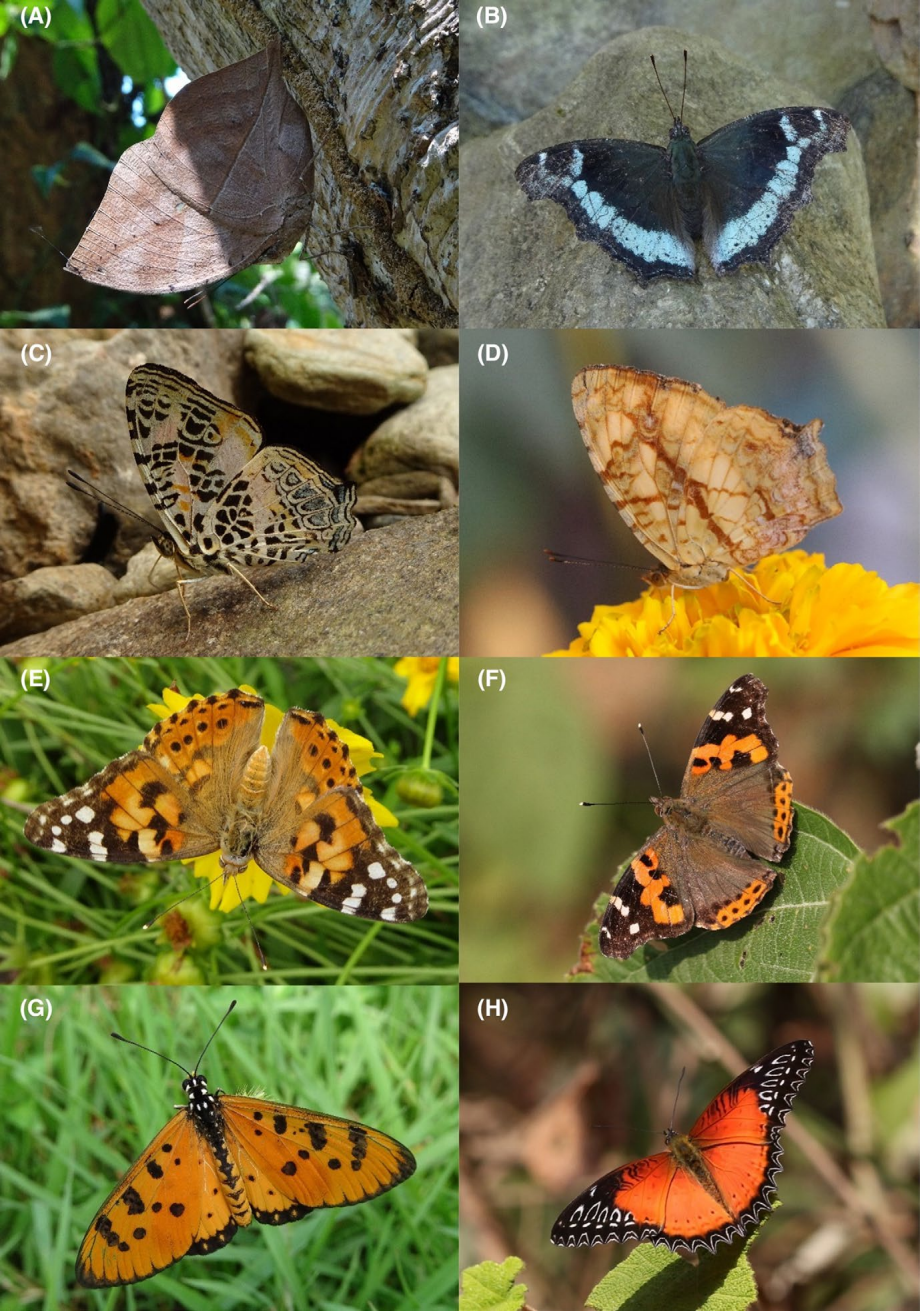
Nymphalidae of Bhorletar. (A) *Kallima inachus inachus* (Boisduval, 1846)—Orange Oakleaf; (B) *Kaniska canace canace* (Linnaeus, 1763)—Blue Admiral; (C) *Symbrenthia hypselis cotanda* Moore, [1875]—Spotted Jester; (D) *Symbrenthia lilaea khasiana* Moore, [1875]—Common Jester; (E) *Vanessa cardui* (Linnaeus, 1758)—Painted Lady; (F) *Vanessa indica indica* (Herbst, 1794)—Indian Red Admiral; (G) *Acraea terpsicore* (Linnaeus, 1758)—Tawny Coster from Sauraha, Chitwan; (H) *Cethosia biblis tisamena* Fruhstorfer, 1912—Red Lacewing from Pokhara, Kaski.

**FIGURE 30:**
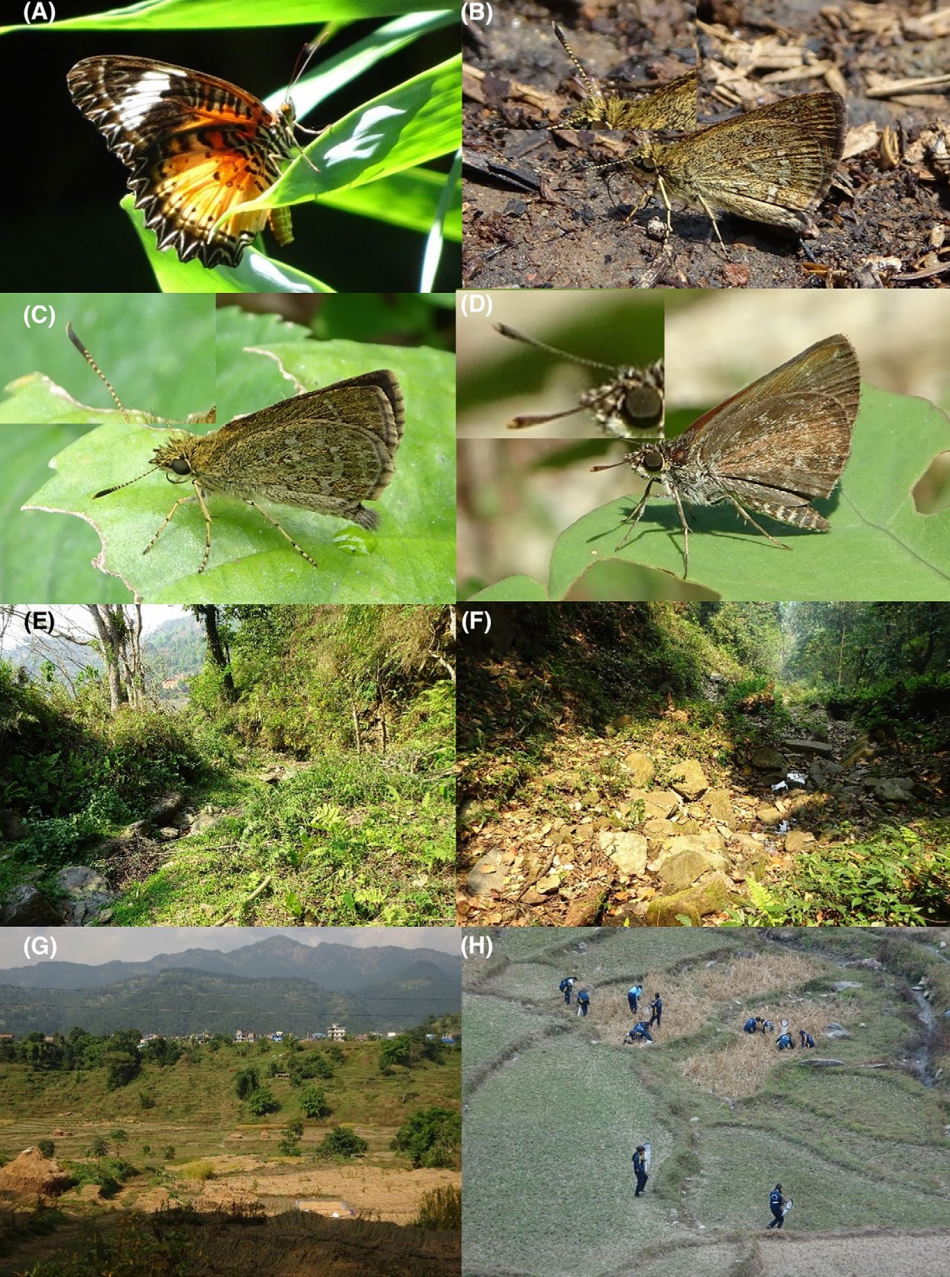
Nymphalidae, cryptic *Aeromachus* (Hesperiidae), and habitat images of Bhorletar. (A) *Cethosia cyane cyane* (Drury, [1773])—Leopard Lacewing; (B) *Aeromachus dubius impha* Evans, 1943—Dingy Scrub Hopper; (C) *Aeromachus jhora jhora* (de Nicéville, 1885)—Grey Scrub Hopper; (D) *Aeromachus pygmaeus* (Fabricius, 1775)—Pygmy Scrub Hopper; (E) Forest stream in April; (F) Forest stream in September; (G) Bhorletar during the harvest season; (H) Students from Ishaneshwor Secondary School, a local government school in Bhorletar, search for insects as part of their assignment.

**FIGURE 31:**
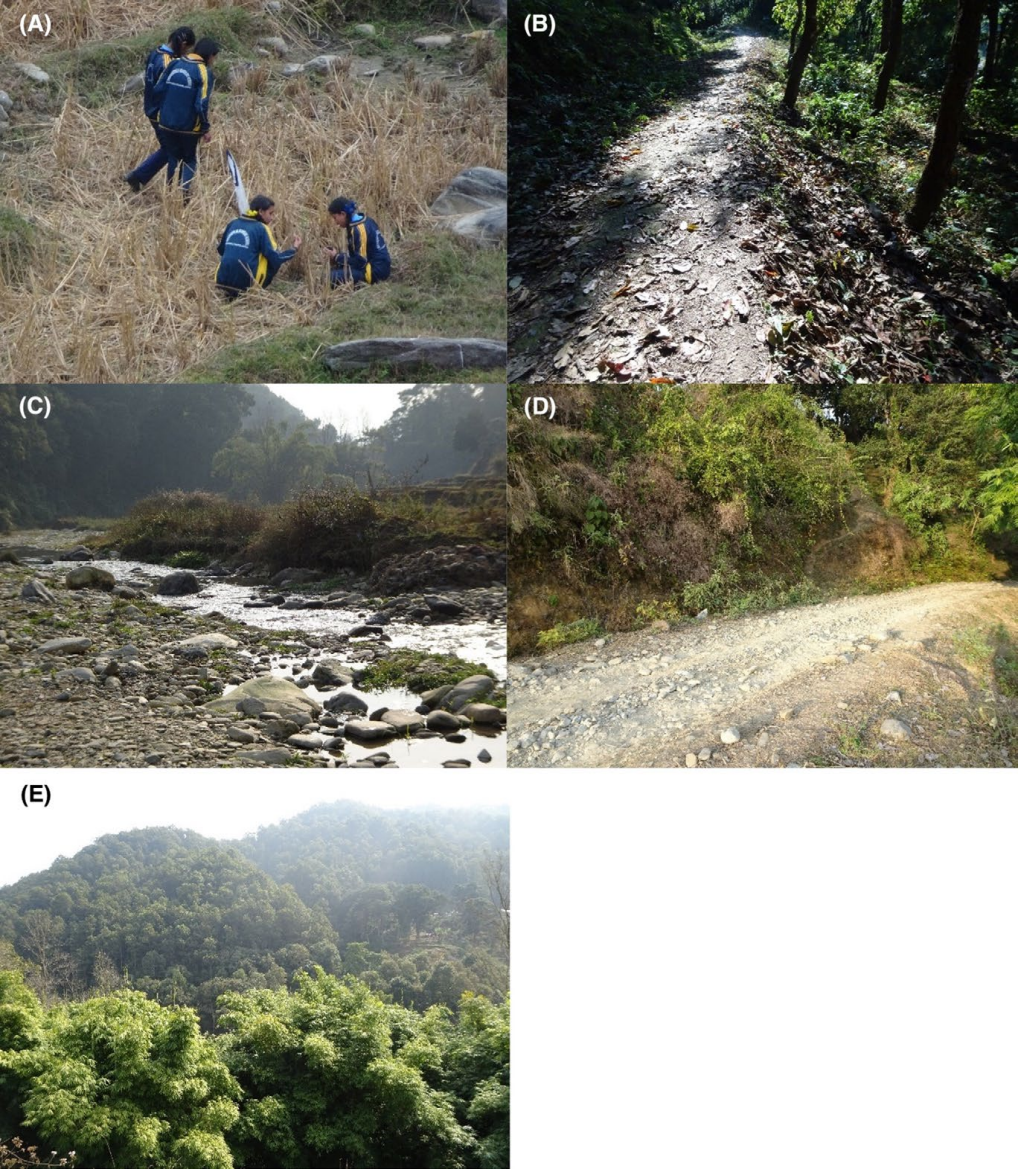
Habitat images of Bhorletar. (A) Students from Ishaneshwor Secondary School, a local government school in Bhorletar, search for insects as part of their assignment; (B) Forest trail in February; (C) Riverside in January; (D) Open trail in January; (E) Local forest in February.

**FIGURE 32:**
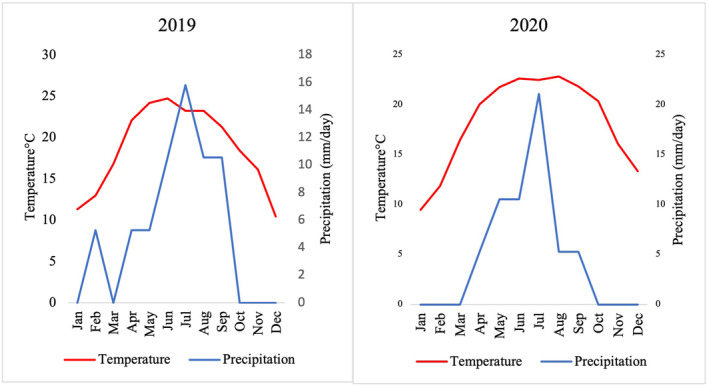
Monthly variation in temperature and precipitation in 2019 and 2020 in Madhya Nepal‐6, Bhorletar, Lamjung, Nepal.

**FIGURE 33:**
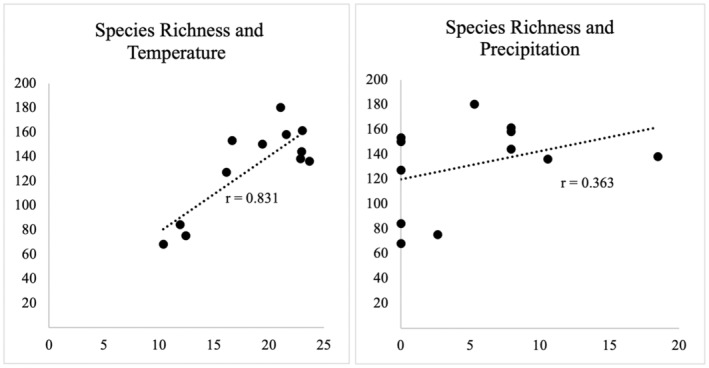
Relationships between the species richness of butterflies with temperature and precipitation in 2019 and 2020 in Madhya Nepal‐6, Bhorletar, Lamjung, Nepal.

A survey of adult butterfly fauna was conducted in the foothills of Bhorletar (28°09'20.1" N, 84°14'15.1" E) (Figure 1) from July 2019 to January 2021 within the elevational range of 420–600 m. The survey was conducted within an approximate 3 km^2^ area. The survey followed an opportunistic design mostly owing to the variable landscape of the study area often altered by seasonal changes. While this method may lack consistency (Van Strien, Van Swaay, and Termaat 2013), such as in following a fixed transect throughout the survey (Pollard 1977, 1979), we maintained uniformity by ensuring an equal number of observation hours each survey session. The survey was conducted six times each month by the first author covering various habitats of the study area, including riverbanks, forest trails, streams, clearings, hilltops, residential areas, and open agricultural fields. Observations were conducted between 10 am and 5 pm Nepal Standard Time (NPT), except when inclement weather or unforeseen circumstances necessitated a pause. Although butterflies could be spotted as soon as temperatures rose, 10 am was deemed the optimal start time for the survey owing to the surge in their activity around this hour (Pollard 1977; Gupta, Tiwari, and Diwakar 2019); evening hours were chosen to align with the flight period of crepuscular skipper species (Chiba 2005, 2009). Any lost hours were made up on subsequent days to ensure comprehensive data collection. Butterflies were photographed in their natural habitats using a Sony Cyber‐Shot DSC‐HX90V camera from 2019 to 2020, and a Canon 7D Mark II camera coupled with a Canon EF 100 mm f/2.8 L Macro IS USM lens from 2020 to 2021. All butterfly and habitat images (Figures 2, 3, 4, 5, 6, 7, 8, 9, 10, 11, 12, 13, 14, 15, 16, 17, 18, 19, 20, 21, 22, 23, 24, 25, 26, 27, 28, 29, 30, 31) presented in this paper were photographed by the first author. The images were automatically geotagged by the cameras. Three species, viz., *Acraea terpsicore* (Linnaeus, 1758), *Cethosia biblis tisamena* Fruhstorfer, 1912, and *Delias hyparete indica* (Wallace, 1867) could not be photographed well during the study and are therefore represented by photographs taken from alternative locations. Specimens that were challenging to identify were collected using a hand net. Following each survey, species counts and abundances were recorded on a survey sheet and subsequently transferred to MS Excel 2019 for data consolidation and monthly analysis. It is important to note that estimates for species with high daily counts (typically exceeding 10 individuals) may not be as accurate as the exact counts possible for species with lower counts (fewer than 10 individuals). To facilitate data presentation, abundance figures have been rounded as follows: tens to the nearest ten, hundreds to the nearest fifty, and counts exceeding 1000 to the nearest hundred. Given the high butterfly abundance in our study area, there is a possibility that some individuals may have been counted multiple times within a day or month. However, to minimize this risk, efforts were made to avoid recounting individuals that had already been observed. Most butterflies were observed engaging in relatively stationary activities such as feeding on flowers, perching on leaves, or mud‐puddling, which helped reduce the likelihood of multiple counts. We also made efforts to avoid recounting individuals that had already been observed, by noting distinctive markings or behaviors. For accurate identification of cryptic species, genitalia analysis was performed using an Olympus Stereo‐microscope Model SZ2‐ILST in the science lab of Ishaneshwor Secondary School, Bhorletar. Genitalia were pretreated by soaking in 10% potassium hydroxide (KOH) solution in Petri dishes for a minimum of 12 h to facilitate dissection and examination. Identifications were made with the help of reference materials such as Evans (1927, 1932, 1949, 1957), Talbot (1947), Kehimkar (2008, 2016), Smetacek (2011, 2015, 2017), Varshney and Smetacek (2015), and online resources (www.flutters.org, www.ifoundbutterflies.org, http://yutaka.it‐n.jp). Previous distribution records in Nepal were compiled from Gough (1935), Bailey (1951), Smith (1994, 2010, 2011a, 2011b), and Van der Poel and Smetacek (2022 first draft). Data analyses were performed using MS Excel 365; the study area map was created using QGIS software (version 3.32.3 “Lima” 2024) and Google Earth (https://earth.google.com). Climatic data, comprising monthly average temperature and precipitation, were obtained from the POWER project (version 2.3.6) (NASA Power 2024) for the study period (Figure 32). A correlation analysis was performed to investigate the relationship between monthly species richness and two key climatic variables: average monthly temperature and precipitation (Figure 33). The Shannon–Wiener index (*H*′) (Shannon and Weaver 1949) (*H*′ = −Σ (*p*_*i*_ * ln(*p*_*i*_)), where Σ represents the summation over all species, *p*_*i*_ is the proportion of individuals belonging to species *i*, and ln is the natural logarithm) was employed to quantify species diversity, encompassing both species richness and abundance, for each family individually and for the overall species assemblage. Additionally, Pielou's *J* index (*J*′) (Pielou 1969) (*J*′ = *H*′/ln(*S*), where *H*′ is the Shannon–Wiener index, and *S* is the total number of species) was utilized to calculate species evenness, which measures the distributional uniformity of species on a scale of 0 to 1, where 1 denotes maximum evenness. Furthermore, Margalef's richness index (*D*) (Margalef 1958) (*D* = (*S* − 1)/ln(*N*), where *S* is the total number of species, and *N* is the total number of individuals) was calculated to estimate species richness, providing a simple yet effective measure of the number of species present in each family while accounting for sample size. The effective number of species (*E*) was determined using the formula *E* = *e* ^(*H*′)^, which employs the Shannon–Wiener index to provide a more nuanced measurement of diversity (Jost 2006). Relative abundance was quantified in percentage for each species recorded, rounded to three decimal places. A local abundance index was developed by the first author to objectively assess the status and rarity of observed species, based on observational data; however, it is important to note that some species, although common in nature, may be elusive and therefore less frequently recorded during surveys. The index categorizes a species as follows:

vF (Very Frequent): Observed in every survey during its flight season.

F (Frequent): Observed more than 10 times but not in every survey during its flight season.

fR (Fairly Rare): Observed 3–10 times throughout the study.

R (Rare): Observed only twice throughout the study.

vR (Very Rare): Observed only once throughout the study.

## 4 Results

**FIGURE 34:**
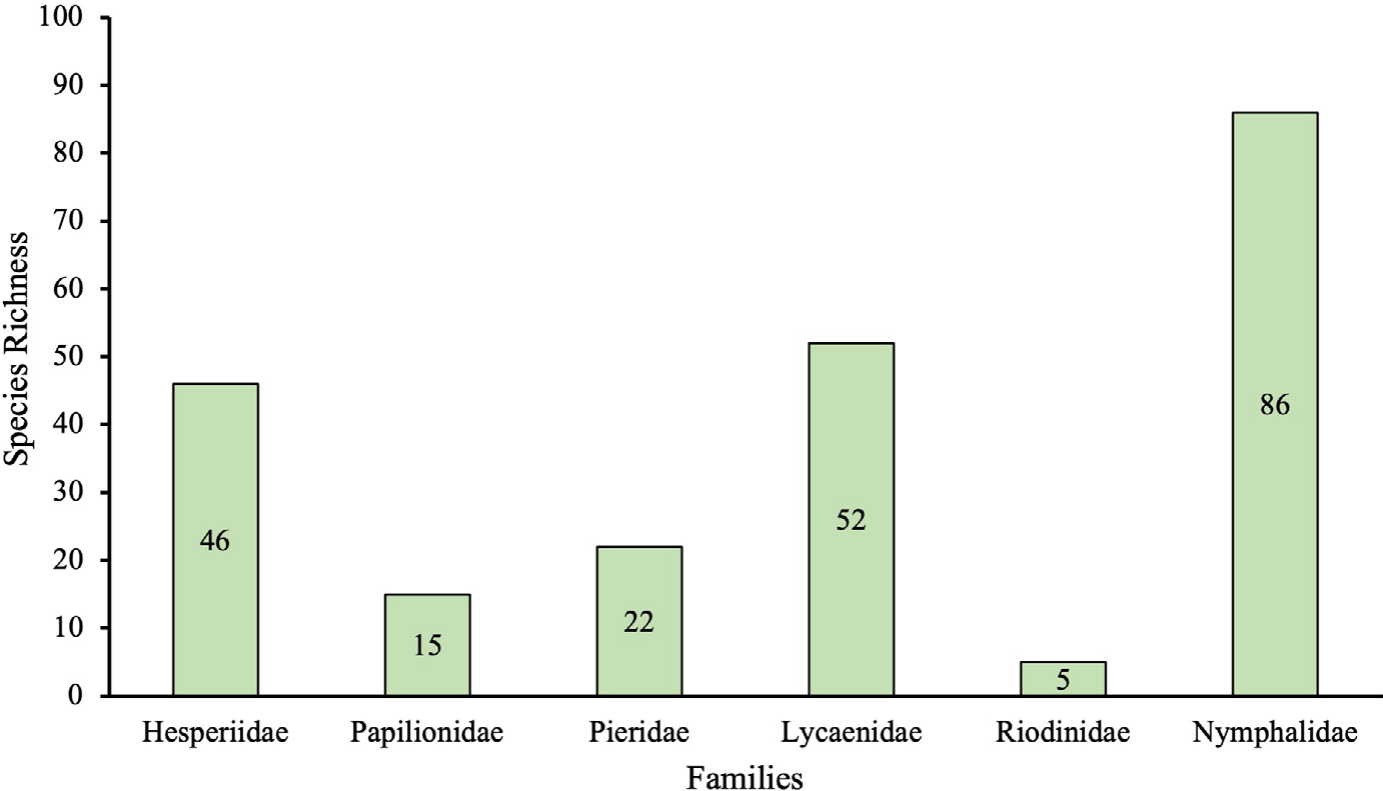
Species richness of butterflies (Lepidoptera: Papilionoidea) across different families as recorded during the study from July 2019 to January 2021 in Madhya Nepal‐6, Bhorletar, Lamjung, Nepal.

**FIGURE 35:**
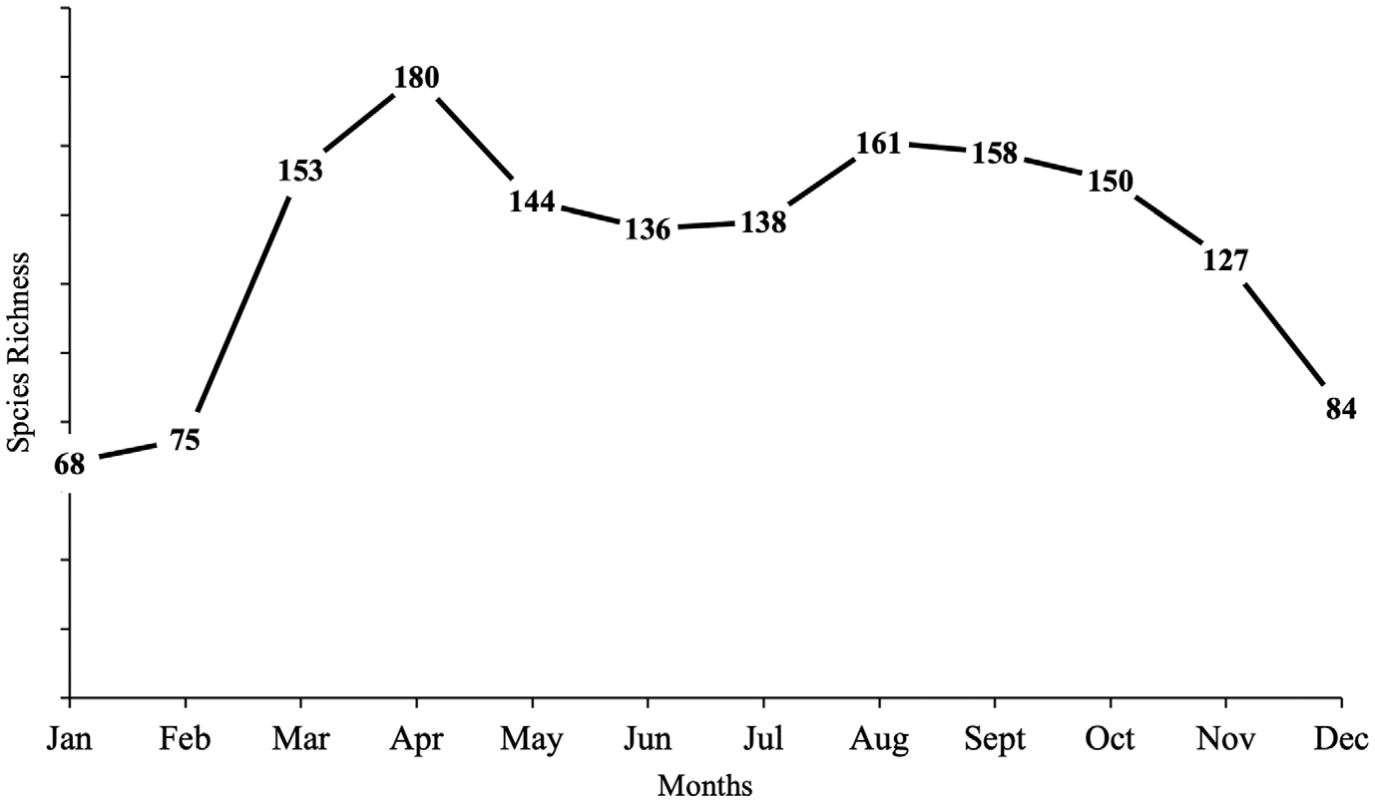
Species richness of butterflies (Lepidoptera: Papilionoidea) by month as recorded during the study conducted from July 2019 to January 2021 in Madhya Nepal‐6, Bhorletar, Lamjung, Nepal.

**FIGURE 36:**
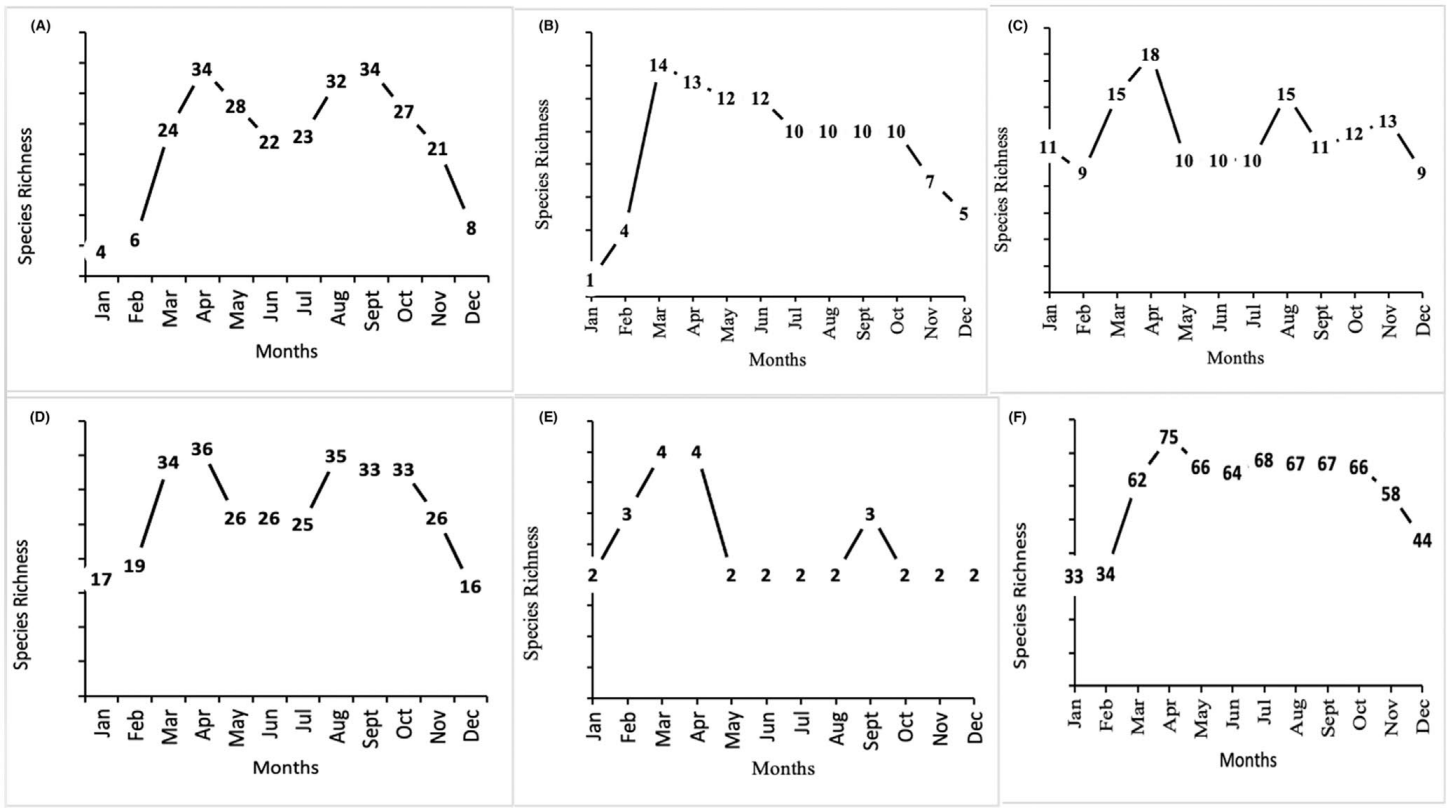
Species richness of butterflies (Lepidoptera: Papilionoidea) by month in different families. (A) Hesperiidae; (B) Papilionidae; (C) Pieridae; (D) Lycaenidae; (E) Riodinidae; (F) Nymphalidae as recorded during the study conducted from July 2019 to January 2021 in Madhya Nepal‐6, Bhorletar, Lamjung, Nepal.

A total of 94,009 individuals across 226 species were recorded, representing 129 genera and six families (Figures 2, 3, 4, 5, 6, 7, 8, 9, 10, 11, 12, 13, 14, 15, 16, 17, 18, 19, 20, 21, 22, 23, 24, 25, 26, 27, 28, 29, 30, 31). Specifically, 86 species were Nymphalidae, 52 species were Lycaenidae, 46 species were Hesperiidae, 22 species were Pieridae, 15 species were Papilionidae, and five species represented Riodinidae (Figure 34). Among the observed species, *Halpe arcuata* (Figure 4F) (see KC 2020) and *Hasora taminatus bhavara* (Figure 1C,D) (see KC and Sapkota 2022) were new to Nepal. *Pantoporia sandaka davidsoni* Eliot, 1969 (Figure 26E) was found to be very common, although it was previously recorded only from Chitwan at the time of the survey (see Sapkota, KC, and Pariyar 2020), and eventually from other places across Nepal (see Van der Poel and Smetacek 2022). This study also documented the second record of *Halpe filda* Evans, 1949 (Figure 4G) in Nepal, previously recorded only once in Sankhuwasabha (eastern Nepal) in 1987 (Smith 1994; Van der Poel and Smetacek 2022). This study also marked the third sighting of *Ctenoptilum vasava vasava* in Nepal (Figure 2H), seen after 1991 (see Van der Poel and Smetacek 2022). Species that are rarely seen in Nepal and documented in this study include *Tarucus waterstradti dharta* Bethune‐Baker, [1918] (Figure 18C), previously recorded only twice (Smith 1994) and never from the central hills of Nepal, *Celaenorrhinus putra putra* (Moore, [1866]) (Figure 2G), previously recorded only from eastern Nepal as very rare (Smith 1994), and *Seseria dohertyi dohertyi* Watson, 1893 (Figure 3D), previously known only from elevations no lower than 1430 m (Van der Poel and Smetacek 2022, first draft). The species identified in this study are detailed in Table 1, which includes their common names, scientific names, observed months, rarities, habitats, and relative abundances in percentage. March through November was found to be the optimal period for recording butterflies in Bhorletar, with March–April and August–September identified as peak months (Figure 35). The month‐by‐month distribution of species within each family is visually represented in Figure 36A–F.

**TABLE 1:** Documented Butterfly Fauna of Bhorletar, Lamjung District, during the Study Period: Scientific and Common Names, Observation Periods, Rarity Status, Habitats, and Relative Abundances. Local Status/Rarity Legend: VF (Very Frequent): Observed in every survey during its flight season; F (Frequent): Observed more than 10 times but not in every survey during its flight season.; fR (Fairly Rare): Observed 3–10 times throughout the study; R (Rare): Observed only twice throughout the study; vR (Very Rare): Observed only once throughout the study.

S.N.	Image number	Common name	Sc. Name	Season	Local status/Rarity	Habitat	Relative abundance (%)
*Family: Hesperiidae* *Subfamily: Coeliadinae*							
1.	2A	Small Green Awlet	*Burara amara* (Moore, [1866])	Aug–Sep	fR	Shady trails	0.006
2.	2B	Branded Orange Awlet	*Burara oedipodea belesis* (Mabille, 1876)	Aug	vR	Forest stream	0.001
3.	2C–D	White‐Banded Awl	*Hasora taminatus bhavara* Fruhstorfer, 1911	Aug–Sep	R	Rural trail, flower	0.002
*Subfamily: Pyrginae*							
4.	2E	Indian Skipper	*Spialia galba galba* (Fabricius, 1793)	Jan–Dec	F	Widespread	0.638
*Subfamily: Tagiadinae*							
5.	2F	Common Spotted Flat	*Celaenorrhinus leucocera* (Kollar, [1844])	Mar–Nov	F	Wildflowers, forest streams	0.425
6.	2G	Bengal Spotted Flat	*Celaenorrhinus putra putra* (Moore, [1866])	Mar–Oct	F	Wildflowers, forest streams	0.064
7.	2H	Tawny Angle	*Ctenoptilum vasava vasava* (Moore, [1866])	Apr	vR	Forest stream	0.001
8.	3A	Dusky Yellow‐Breasted Flat	*Gerosis phisara phisara* (Moore, 1884)	Mar–Apr, Oct	fR	Forest streams	0.006
9.	3B	Fulvous Pied Flat	*Pseudocoladenia fatih* (Kollar, [1844])	Mar–Dec	F	Forest streams, flowers	0.425
10.	3C	Common Small Flat	*Sarangesa dasahara dasahara* (Moore, [1866])	Jan–Dec	vF	Widespread	1.170
11.	3D	Himalayan White Flat	*Seseria dohertyi dohertyi* Watson, 1893	Apr	vR	Forest stream	0.001
12.	3E	Suffused Snow Flat	*Tagiades gana athos* Plötz, 1884	Mar–Dec	F	Shady trails, forest streams, riversides	0.372
13.	3F	Water Snow Flat	*Tagiades litigiosa litigiosa* Fruhstorfer, 1910	Mar–Dec	vF	Shady trails, forest streams, riversides	0.745
14.	3G	Spotted Snow Flat	*Tagiades menaka menaka* (Moore, [1866])	Mar–Nov	vF	Shady trails, forest streams	0.638
15.	3H	Large Snow Flat	*Tagiades parra gala* Evans, 1949	Mar–Oct	F	Shady trails, forest streams	0.266
*Subfamily: Hesperiinae*							
16.	30B	Dingy Scrub Hopper	*Aeromachus dubius impha* Evans, 1943	Apr–Oct	vF	Widespread	0.239 (mixed with the next species)
17.	30C	Grey Scrub Hopper	*Aeromachus jhora jhora* (de Nicéville, 1885)	Apr–Oct	vF	Widespread	0.239
18.	30D	Pygmy Scrub Hopper	*Aeromachus pygmaeus* (Fabricius, 1775)	Sep	vR	Open country	0.001
19.	4A	Paintbrush Swift	*Baoris farri farri* (Moore, 1878)	Mar–Oct	F	Forest, forest streams	0.160
20.	4B	Rice Swift	*Borbo cinnara* (Wallace, 1866)	Aug, Nov	fR	Grasslands, flowers, open country	0.007
21.	4C	Colon Swift	*Caltoris cahira austeni* (Moore, [1884])	Mar–Oct	F	Forest, forest streams	0.266
22.	4D	Blank Swift	*Caltoris kumara moorei* (Evans, 1926)	Mar, Apr, Sep	fR	Forest, forest streams	0.008
23.	4E	Sikkim Palm Red Eye	*Erionota torus* Evans, 1941	Aug, Sep	F	Forest streams, shady tree trunks	0.042
24.	4F	Overlapped Ace	*Halpe arcuata* Evans, 1937	Apr–May, Nov	F–very local	Forest streams	0.042 (some individuals could have been the next species)
25.	4G	Elwes' Ace	*Halpe filda* Evans, 1949	Apr–May	fR–very local	Forest streams	0.004 (likely more, mixed with the previous species)
26.	4H	Chestnut Bob	*Iambrix salsala salsala* (Moore, [1866])	Apr–Nov	vF	Forest, wildflowers	0.691
27.	5A	Common Red Eye	*Matapa aria* (Moore, [1866])	Mar–Nov	vF	Forest streams, shady trails	0.745
28.	5B	Grey‐Brand Red Eye	*Matapa druna* (Moore, [1866])	Aug	vR	Shady trail	0.001
29.	5C	Black–Veined Red Eye	*Matapa sasivarna* (Moore, [1866])	Sep	vR	Shady forest stream	0.001
30.	5D	Restricted Demon	*Notocrypta curvifascia curvifascia* (C. & R. Felder, 1862)	Mar–Nov	vF	Forest streams, riversides	0.691
31.	5E	Ceylon Dartlet	*Oriens goloides* (Moore, [1881])	Apr–May	fR	Flower, forest stream	0.004
32.	5F	Sumatran Swift	*Parnara apostata debdasi* Chiba & Eliot, 1991	Feb–May, Aug–Oct	fR	Flowers, open country	0.004
33.	5G	Ceylon Swift	*Parnara bada bada* (Moore, 1878)	Jan–Dec	vF	Flowers, open country	0.957
34.	5H	Straight Swift	*Parnara guttatus mangala* (Moore, [1866])	Mar–May, Aug–Oct	F	Flowers, open country	0.479
35.	6A	Small Branded Swift	*Pelopidas mathias mathias* (Fabricius, 1798)	Jan–Dec	vF	Grassland, open country, flowers	1.010
36.	6B	Little Branded Swift	*Pelopidas agna agna* (Moore, [1866])	Aug–Oct	F	Grassland, open country, flowers	0.160
37.	6C	Great Swift	*Pelopidas assamensis* (de Nicéville, 1882)	Sept–Nov	fR	Forest streams	0.003
38.	6D	Light Straw Ace	*Pithauria stramineipennis stramineipennis* Wood–Mason & de Nicéville, [1887]	Mar–Jul, Nov	F	Riversides, forest streams	0.053
39.	6E	Pale Dart	*Potanthus pallida* (Evans, 1932)	Apr	fR	Forest streams	0.004
40.	6F	Indian Dart	*Potanthus pseudomaesa clio* (Evans, 1932)	Mar–Nov	vF	Forest streams, open trails, wildflowers	0.851
41.	6G–H	Bevan's Swift	*Pseudoborbo bevani* (Moore, 1878)	Feb–Dec	vF	Open country, grassland, flowers	1.170
42.	7A	Common Grass Dart	*Taractrocera maevius sagara* (Moore, [1866])	Jul–Sep	F	Grasslands, open trails	0.106
43.	7B	Dark Palm Dart	*Telicota bambusae* (Moore, 1878)	Mar–Oct	vF	Flowers, forest streams, open trails	0.745
44.	7C	Plotz's Palm Dart	*Telicota ohara jix* Evans, 1949	Apr, Sep, Nov	fR	Forest streams	0.004
45.	7D	Grass Demon	*Udaspes folus* (Cramer, [1775])	Mar–Oct	F	Flowers, open trails	0.106
46.	7E	Himalayan Swift	*Zenonoida discreta discreta* (Elwes & Edwards, 1897)	Nov	vR	Forest stream	0.001
*Family: Papilionidae* *Subfamily: Papilioninae*							
47.	7F	Tailed Jay	*Graphium agamemnon agamemnon* (Linnaeus, 1758)	Feb–Dec	vF	Open country, forests, forest streams, riversides	0.851
48.	7G	Glassy Bluebottle	*Graphium cloanthus cloanthus* (Westwood, 1841)	Mar–May	F	Riversides	0.042
49.	7H	Common Jay	*Graphium doson axionides* (Page & Treadaway, 2014)	Mar	fR	Riversides	0.004
50.	8A	Common Bluebottle	*Graphium sarpedon sarpedon* (Linnaeus, 1758)	Feb–Oct	vF	Open country, flowers, riversides	0.745
51.	8B	Common Rose	*Pachliopta aristolochiae aristolochiae* (Fabricius, 1775)	Mar–Oct	F	Open country, flowers, riversides	0.064
52.	8C	Common Peacock	*Papilio bianor ganesa* Moore, 1842	Apr–Jun	fR	Flowers	0.003
53.	8D	Common Mime	*Papilio clytia clytia* Linnaeus, 1758	Mar–Oct	F	Flowers, riversides	0.053
54.	8E	Lime Swallowtail	*Papilio demoleus demoleus* Linnaeus, 1758	Jan–Dec	vF	Citrus plants, riversides, flowers	1.064
55.	8F	Lesser Mime	*Papilio epycides epycides* Hewitson, 1864	Mar–Apr	fR	Riversides	0.003
56.	8G	Red Helen	*Papilio helenus helenus* Linnaeus, 1758	Mar–Nov	vF	Riversides, shady trails, forest streams	0.798
57.	8H	Great Mormon	*Papilio memnon agenor* Linnaeus, 1758	Mar–Nov	vF	Riversides, citrus plants, flowers	0.851
58.	9A	Yellow Helen	*Papilio nephelus chaon* Westwood, 1844	Mar–Dec	F	Riversides, shady trails, forest streams	0.064
59.	9B	Paris Peacock	*Papilio paris paris* Linnaeus, 1758	Mar–Nov	vF	Riversides, open country, flowers, forest streams, open trails	0.745
60.	9C	Common Mormon	*Papilio polytes romulus* Cramer, [1775]	Feb–Dec	vF	Widespread	1.064
61.	9D	Spangle	*Papilio protenor euprotenor* Fruhstorfer, 1908	Mar, Jun	fR	Citrus plants, forest flowers in sunny places	0.004
*Family: Pieridae* *Subfamily: Coliadinae*							
62.	9E	Common Emigrant	*Catopsilia pomona pomona* (Fabricius, 1775)	Jan–Dec	vF	Open country, flowers, agricultural fields, open trails, riversides	1.276
63.	9F	Mottled Emigrant	*Catopsilia pyranthe pyranthe* (Linnaeus, 1758)	Jan–Dec	vF	Open country, flowers, agricultural fields, riversides, open trails	1.170
64.	9G	Dark Clouded Yellow	*Colias fieldii fieldii* Ménétriés, 1855	Jan–Dec	F	Agricultural fields, open country, grasslands	0.425
65.	9H	Three‐Spot Grass Yellow	*Eurema blanda silhetana* (Wallace, 1867)	Jan–Dec	vF	Widespread, mostly on flowers and riversides	1.489
66.	10A	Common Grass Yellow	*Eurema hecabe hecabe* (Linnaeus, 1758)	Jan–Dec	vF	Widespread, mostly on flowers and riversides	1.915
67.	10B	Spotless Grass Yellow	*Eurema laeta laeta* (Boisduval, 1836)	Jan–Dec	vF	Open trails	1.702
68.	10C	Tree Yellow	*Gandaca harina assamica* Moore, [1906]	Apr, Aug	vF	Forest streams, riversides, females mostly on flowers	0.085
*Subfamily: Pierinae*							
69.	10D	Chocolate Albatross	*Appias lyncida eleonora* (Boisduval, 1836)	Jan, Apr–Sep, Nov–Dec	vF	Widespread except shady trails	0.425
70.	10E	Lesser Gull	*Cepora nadina nadina* (Lucas, 1852)	Feb–Apr, Aug, Nov	vF	Riversides, flowers, forest, forest streams	0.213
71.	10F	Common Gull	*Cepora nerissa phryne* (Fabricius, 1775)	Jan, Mar, Apr, Aug	F	Riversides, forest, forest streams, flowers	0.064
72.	10G	Red‐Breast Jezebel	*Delias acalis pyramus* (Wallace, 1867)	Mar	vR	Riverside	0.001
73.	10H	Hill Jezebel	*Delias belladonna horsfieldi* (Gray, 1831)	Apr	R	Forest stream	0.002
74.	11A	Common Jezebel	*Delias eucharis* (Drury, 1773)	Dec	vR	Flower	0.001
75.	11B	Painted Jezebel	Delias hyparete indica (Wallace, 1867)	Apr, Aug–Oct	vF	Forest streams, flowers	0.479
76.	11C	Red‐Base Jezebel	*Delias pasithoe dione* (Drury, [1773])	Jan, Aug–Nov	vF	Agricultural fields, flowers, riversides	0.532
77.	11D	Great Orange Tip	*Hebomoia glaucippe glaucippe* (Linnaeus, 1758)	Mar–Apr, Oct–Nov	F	Riversides, forest streams	0.064
78.	11E	Yellow Orange Tip	*Ixias pyrene latifasciata* (Fabricius, 1777)	Jan, Mar–Apr, Oct–Nov	vF	Riversides, flowers, forest streams	0.851
79.	11F	Pale Wanderer	*Pareronia avatar* (Moore, [1858])	Apr, Aug–Nov	fR	Forest streams	0.004
80.	11G	Large Cabbage White	*Pieris brassicae nepalensis* Gray, 1846	Feb–Oct	F	Agricultural fields, open trails, flowers	0.638
81.	11H	Indian Cabbage White	*Pieris canidia indica* Evans, 1926	Jan–Dec	vF	Agricultural fields, open trails, flowers	2.553
82.	12A	Bath White	*Pontia daplidice moorei* (Röber, [1907])	Mar–May, Jul	vF	Agricultural fields, open trails, flowers	0.213
83.	12B	Spotted Sawtooth	*Prioneris thestylis thestylis* (Doubleday, 1842)	Mar, Jun	fR	Riversides, open trails	0.004
*Family: Lycaenidae* *Subfamily: Curetinae*							
84.	12C	Bright Sunbeam	*Curetis bulis bulis* (Westwood, 1852)	Apr, Aug, Oct, Nov	F	Riversides, forest streams	0.085
*Subfamily: Poritinae*							
85.	12D	Common Gem	*Poritia hewitsoni hewitsoni* Moore, [1866]	Aug	vR	Open trail	0.001 (1 dead individual)
*Subfamily: Miletinae*							
86.	12E	Forest Pierrot	*Taraka hamada mendesia* Fruhstorfer, 1918	Mar	fR	Forest	0.004
*Subfamily: Lycaeninae*							
87.	12F	Purple Sapphire	*Heliophorus epicles latilimbata* Eliot, 1963	May, Aug–Oct	vF	Forest, forest trails, forest streams, riversides	0.425 (mixed with the next species)
88.	12G	Indian Purple Sapphire	*Heliophorus indicus* (Fruhstorfer, 1908)	Mar–Apr, Aug–Oct	vF	Forest trails, forest, riversides, forest streams	0.425
*Subfamily: Aphnaeinae*							
89.	12H	Long‐Banded Silverline	*Spindasis lohita himalayanus* (Moore, 1884)	Apr–May, Sep	fR	Flowers, forest streams	0.004
90.	13A	Club Silverline	*Spindasis syama peguanus* Moore, 1884	Apr, Aug, Sep	F	Flowers, forest streams	0.042
*Subfamily: Theclinae*							
91.	13B	Centaur Oakblue	*Arhopala centaurus pirithous* (Moore, [1884])	Jan–Dec	vF	Forest streams, riversides, forest	0.957
92.	13C	Green Oakblue	*Arhopala eumolphus eumolphus* (Cramer, [1780])	Apr	fR	Forest stream	0.004
93.	13D	Hewitson's Dull Oakblue	*Arhopala oenea* (Hewitson, 1869)	Jan–Apr, Oct	F	Forest trails, forest	0.160
94.	13E	Hooked Oakblue	*Arhopala paramuta paramuta* (de Nicéville, [1884])	Feb–Nov	vF	Forest trails, forest	1.064
95.	13F	Common Tinsel	*Catapaecilma major major* Druce, 1895	Mar–Apr, Aug	F	Forest streams, rarely riversides	0.053
96.	13G	Orchid Tit	*Chliaria othona othona* (Hewitson, 1865)	Apr–Jun, Sep–Oct	vF	Forest streams	0.160
97.	16F	Common Onyx	*Horaga onyx onyx* (Moore, 1858)	Mar	R	Forest trail	0.002
98.	13H	Common Tit	*Hypolycaena erylus himavantus* Fruhstorfer, 1912	Sep–Nov	vF	Forest streams, open trails	0.160
99.	14A	Silverstreak Blue	*Iraota timoleon timoleon* (Stoll, [1790])	Aug–Sep	fR	Tree leaves	0.008
100.	14B	Yamfly	*Loxura atymnus atymnus* (Stoll, 1780)	Mar, Aug–Oct	fR	Riversides, forest streams	0.003
101.	14C	Slate Flash	*Rapala manea schistacea* (Moore, 1879)	Jan–Dec	F	Open country, *Rubus* L. leaves, riversides	0.160
102.	14D	Common Flash	*Rapala nissa nissa* (Kollar, [1844])	Aug	vR	Forest stream	0.001
103.	14E	Copper Flash	*Rapala pheretima petosiris* (Hewitson, 1863)	Mar, Apr–Aug	F	Wildflowers, forest streams, forest	0.064
104.	14F	Shot Flash	*Rapala* cf. *rectivitta* (Moore, 1879)	Feb–Apr	vF	Forest trails, flowers, forest streams	0.106
105.	14G	Indigo Flash	*Rapala varuna gebenia* Fruhstorfer, 1914	Mar–Apr	fR	Forest trails	0.010
106.	14H	Broad Spark	*Sinthusa chandrana chandrana* (Moore, 1882)	Feb–Apr, Jun–Oct	F	Forest trails	0.160
107.	15A	Common Acacia Blue	*Surendra quercetorum quercetorum* (Moore, [1858])	Jan–Mar, Jun–Aug, Nov	F	Forest trails, forest streams, open trails	0.266
108.	15B	Blue Imperial	*Ticherra acte acte* (Moore, [1858])	Apr	fR	Forest stream	0.006
109.	15C	Fluffy Tit	*Zeltus amasa amasa* (Hewitson, 1865)	Jan, Apr, Oct–Nov	vF	Forest streams, riversides	0.266
*Subfamily: Polyommatinae*							
110.	15D	Common Hedge Blue	*Acytolepis puspa gisca* (Fruhstorfer, 1910)	Jan–Dec	vF	Forest streams, riversides	0.957
111.	15E	Ciliate Blue	*Anthene emolus emolus* (Godart, [1824])	Mar–Nov	vF	Forest streams, riversides	1.064
112.	15F	Elbowed Pierrot	*Caleta elna noliteia* (Fruhstorfer, 1918)	Aug–Nov	F	Open/shady trails, forest streams	0.074
113.	15G	Common Pierrot	*Castalius rosimon rosimon* (Fabricius, 1775)	Jan–Dec	vF	Open trails, riversides, *Ziziphus* Mill. Plants	1.170
114.	15H	Forget–Me–Not–Blue	*Catochrysops strabo strabo* (Fabricius, 1793)	Mar, Nov	fR	Open trail, flowers	0.004
115.	16A	Plain Hedge Blue	*Celastrina lavendularis limbata* (Moore, 1879)	May–Jun	fR	Open trail	0.003
116.	16B	Margined Hedge Blue	*Celatoxia marginata* (de Nicéville, 1884)	Sept	R	Forest streams, riverside forest	0.002
117.	16C	Lime Blue	*Chilades lajus lajus* (Stoll, [1780])	Jul–Oct	F	Open trails	0.064
118.	16D	Indian Cupid	*Cupido lacturnus assamica* Tytler, 1915	Mar–Oct	F	Open trails, grasslands	0.372
119.	16E	Gram Blue	*Euchrysops cnejus cnejus* (Fabricius, 1798)	Jan–Dec	vF	Open trails, grasslands, agricultural gardens	1.064
120.	16G	Metallic Cerulean	*Jamides alecto alocina* Swinhoe, 1915	Jan–Dec	vF	Forest trails, forest, riversides, forest streams	0.851
121.	16H	Dark Cerulean	*Jamides bochus bochus* (Stoll, [1782])	Jan–Dec	vF	Forest trails, forest, riversides, forest streams	0.851
122.	17A	Common Cerulean	*Jamides celeno aelianus* (Fabricius, 1793)	Jan–Dec	vF	Forest trails, forest, riversides, forest streams, open trails	0.957
123.	17B	Pea Blue	*Lampides boeticus* (Linnaeus, 1767)	Jan–Dec	F	Agricultural fields, riversides, open trails	0.425
124.	17C	White‐Banded Hedge Blue	*Lestranicus transpectus* (Moore, 1879)	Mar–Dec	F	Forest streams, forest trails, riversides	0.266
125.	17D	Malayan	*Megisba malaya sikkima* Moore, 1884	Apr–Jul	F	Forest streams	0.053
126.	17E	Transparent Sixline‐Blue	*Nacaduba kurava euplea* Fruhstorfer, 1916	Mar	vR	Forest	0.001
127.	17F	Tailless Lineblue	*Prosotas dubiosa indica* (Evans, [1925])	Aug, Oct	R	Forest streams	0.002
128.	17G	Common Lineblue	*Prosotas nora nora* (C. Felder, 1860)	Jan–Dec	F	Riversides	0.425
129.	17H	Margined Lineblue	*Prosotas pia marginata* Tite, 1963	Jan–Dec	vF	Riversides	1.276
130.	18A	Pale Grass Blue	*Pseudozizeeria maha maha* (Kollar, [1844])	Jan–Dec	vF	Open country, grasslands, agricultural fields, flowers	2.127
131.	18B	Dark Pierrot	*Tarucus ananda* (de Nicéville, [1884])	Mar–Dec	F	Riversides, forest streams	0.042
132.	18C	Assam Pierrot	*Tarucus waterstradti dharta* Bethune‐Baker, [1918]	Apr, Jun, Nov	fR	Forest streams, forest trails	0.006
133.	18D	Pale Hedge Blue	*Udara dilecta dilecta* (Moore, 1879)	Jan–Dec	vF	Forest streams, riversides	1.170
134.	18E	Dark Grass Blue	*Zizeeria karsandra* (Moore, 1865)	Mar–Oct	vF	Agricultural fields, riversides	0.745
135.	18F	Lesser Grass Blue	*Zizina otis indica* (Murray, 1874)	Jan–Dec	vF	Open country, grasslands, agricultural fields, flowers	1.276
*Family: Riodinidae* *Subfamily: Nemeobiinae*							
136.	18G	Spot Judy	*Abisara chela chela* de Nicéville, 1886	Feb–Apr	fR	Riversides, open trails	0.004
137.	18H	Dark Judy	*Abisara fylla* (Westwood, 1851)	Jan–Dec	vF	Forest trails, forest, riversides	1.489
138.	19A	Tailed Judy	*Abisara neophron neophronides* Fruhstorfer, 1914	Apr, Sept	R	Forest trail, forest	0.002
139.	19B	Orange Punch	*Dodona egeon egeon* (Westwood, [1851])	Mar	vR	Forest stream	0.001
140.	19C	Punchinello	*Zemeros flegyas indicus* Fruhstorfer, 1898	Jan–Dec	vF	Forest, forest trails, open trails, shady trails	1.596
*Family: Nymphalidae* *Subfamily: Danainae*							
141.	19D	Plain Tiger	*Danaus chrysippus chrysippus* (Linnaeus, 1758)	Jan–Dec	vF	Flowers, riversides	1.276
142.	19E	Common Tiger	*Danaus genutia genutia* (Cramer, [1779])	Jan–Dec	vF	Flowers, riversides	1.170
143.	19F	Common Crow	*Euploea core core* (Cramer, [1780])	Jan–Dec	vF	Flowers, riversides	1.276
144.	19G	Brown King Crow	*Euploea klugii kollari* C. & R. Felder, [1865]	Jul	vR	Open trail	0.001
145.	19H	Striped Blue Crow	*Euploea mulciber mulciber* (Cramer, [1777])	Jan–Dec	vF	Riversides, around trees	0.745
146.	20A	Glassy Tiger	*Parantica aglea melanoides* Moore, 1883	Jan–Dec	vF	Riversides, forest, forest streams	1.064
147.	20B	Chocolate Tiger	*Parantica melaneus plataniston* (Fruhstorfer, 1910)	May	fR	Riversides, open trail	0.003
148.	20C	Blue Tiger	*Tirumala limniace exoticus* (Gmelin, 1790)	Apr–May	F	Flowers, open trails	0.042
149.	20D	Dark Blue Tiger	*Tirumala septentrionis septentrionis* (Butler, 1874)	Jan–Dec	vF	Riversides, open trails, forest streams, flowers	0.745
*Subfamily: Charaxinae*							
150.	20E	Tawny Rajah	*Charaxes bernardus hierax* C. & R. Felder, [1867]	Mar–Nov	F	Riversides, forest streams, open trails, feeding on excreta or carcasses	0.042
151.	20F	Common Nawab	*Polyura athamas athamas* (Drury, [1773])	Mar–Nov	F	Riversides, forest streams, open trails, feeding on excreta or carcasses	0.085
*Subfamily: Amathusinae*							
152.	20G	Common Duffer	*Discophora sondaica zal* Westwood, 1851	Mar–Nov	vF	Shady trails under bamboo clumps	0.425
Subfamily: Satyrinae							
153.	20H	Spotted Palmfly	*Elymnias malelas malelas* (Hewitson, 1863)	Mar–Oct	vF	Shady trails, riversides, forest streams	0.319
154.	21A	Jezebel Palmfly	*Elymnias vasudeva vasudeva* Moore, 1857	Apr–Jul	fR	Forest streams	0.004
155.	21B	White‐Line Bushbrown	*Heteropsis malsara* (Moore, 1857)	Jan–Dec	F	Shady trails, open trails, bamboo clumps	0.425
156.	21C	Angled Red Forester	*Lethe chandica chandica* (Moore, [1858])	Mar–Nov	vF	Bamboo clumps, shady trails	0.532
157.	21D	Banded Treebrown	*Lethe confusa confusa* Aurivillius, 1898	Mar–Dec	vF	Bamboo clumps, shady trails, riversides	0.957
158.	21E	Scarce Red Forester	*Lethe distans* Butler, 1870	Apr	vR	Bamboo clumps	0.001
159.	21F	Bamboo Treebrown	*Lethe europa niladana* Fruhstorfer, 1911	Apr–Nov	F	Bamboo clumps, shady trails	0.319
160.	21G	Bamboo Forester	*Lethe kansa kansa* (Moore, 1857)	Apr	vR	Forest stream	0.001
161.	21H	Common Red Forester	*Lethe mekara mekara* (Moore, [1858])	Mar–Nov	F	Bamboo clumps, shady trails	0.213
162.	22A	Common Evening Brown	*Melanitis leda leda* (Linnaeus, 1758)	Jan–Dec	vF	Paddy fields, grasslands, forest	0.957
163.	22B	Dark Evening Brown	*Melanitis phedima bela* Moore, 1857	Jan–Dec	F	Paddy fields, grasslands, forest	0.266
164.	22C	Watson's Bushbrown	*Mycalesis adamsoni adamsoni* Watson, 1897	Apr–Oct	F	Forest, shady trails	0.425
165.	22D	Dark‐Brand Bushbrown	*Mycalesis mineus mineus* (Linnaeus, 1758)	Jan–Dec	vF	Paddy fields, forest, shady trails	1.170
166.	22E	Common Bushbrown	*Mycalesis perseus blasius* (Fabricius, 1798)	Jan–Dec	F	Paddy fields, forest, shady trails	0.213
167.	22F	Long‐Brand Bushbrown	*Mycalesis visala visala* Moore, [1858]	Jan–Dec	F	Shady trails, open trails, riversides	0.372
168.	22G	Jungle Brown	*Orsotriaena medus medus* (Fabricius, 1775)	Jan–Dec	vF	Paddy fields, forest, shady trails, riversides, grasslands	1.596
169.	22H	Jewel Fivering	*Ypthima avanta* Moore, [1875]	Jul	vR	Grasslands	0.001 (probably more common but overlooked)
170.	23A	Common Fivering	*Ypthima baldus baldus* (Fabricius, 1775)	Jan–Dec	vF	Grasslands, open trails	1.702
171.	23B	Common Fourring	*Ypthima huebneri* Kirby, 1871	Jan–Dec	vF	Grasslands, open trails	1.702
172.	23C	Brown Argus	*Ypthima hyagriva nepalica* Smith, 1983	Sept	R	Forest stream, open country	0.002
173.	23D	Newar Threering	*Ypthima newara newara* Moore, [1875]	Jul–Oct	F	Forest streams	0.074
*Subfamily: Limenitidinae*							
174.	23E	Sergeant Major	*Abrota ganga ganga* Moore, 1857	Nov	vR	Forest stream	0.001
175.	23F	Orange Staff Sergeant	*Athyma cama cama* Moore, [1858]	Mar–Nov	F	Forest streams	0.213
176.	23G	Color Sergeant	*Athyma nefte inara* (Westwood, 1850)	Feb–Dec	vF	Forest streams, riversides	0.532
177.	23H	Oriental Sergeant	*Athyma orientalis* Elwes, 1888	Apr, Nov	fR	Forest streams	0.004
178.	24A	Common Sergeant	*Athyma perius perius* (Linnaeus, 1758)	Apr	vR	Riverside	0.001
179.	24B	Blackvein Sergeant	*Athyma ranga ranga* Moore, [1858]	Apr–Dec	F	Forest streams, riversides	0.106
180.	24C	Staff Sergeant	*Athyma selenophora selenophora* (Kollar, [1844])	Mar–Dec	vF	Forest streams, riversides	0.532
181.	24D	Small Staff Sergeant	*Athyma zeroca zeroca* Moore, 1872	Mar–Nov	F	Forest streams, riversides	0.213
182.	24E	Grey Count	*Cynitia lepidea lepidea* (Butler, 1868)	Mar–Dec	vF	Forest streams, riversides, open/shady trails	1.276
183.	24F	Common Baron	*Euthalia aconthea suddhodana* Fruhstorfer, 1913	Mar–Dec	F	Forest streams, open trails, forest trails	0.213
184.	24G	Powdered Baron	*Euthalia monina kesava* (Moore, 1859)	Jul–Aug	fR	Forest trails	0.008
185.	24H	Yellowjack Sailer	*Lasippa viraja viraja* (Moore, 1872)	Mar–Oct	F–very local	Forest, forest stream	0.042
186.	25A	Commander	*Moduza procris procris* (Cramer, [1777])	Jul, Nov, Dec	fR	Riversides, forest streams, open trails	0.008
187.	25B	Yellow Sailer	*Neptis ananta ochracea* Evans, 1924	Apr	R	Forest stream	0.002
188.	25C	Plain Sailer	*Neptis cartica cartica* Moore, 1872	Mar–Oct	F	Forest streams, riversides	0.064
189.	25D	Sullied Sailer	*Neptis clinia susruta* Moore, 1872	Mar–Dec	vF	Forest streams, riversides, open trails	0.691
190.	25E	Common Sailer	*Neptis hylas kamarupa* Moore, [1875]	Jan–Dec	vF	Forest streams, riversides, open trails, grasslands	1.489
191.	25F	Spotted Sailer	*Neptis magadha khasiana* Moore, 1872	Mar–Oct	F	Forest streams, open trails	0.064
192.	25G	Small Yellow Sailer	*Neptis miah miah* Moore, 1857	Apr	vR	Forest stream	0.001
193.	25H	Clear Sailer	*Neptis nata adipala* Moore, 1872	Mar–Nov	F	Forest, forest streams, riversides	0.372
194.	26A	Broad‐Banded Sailer	*Neptis sankara amba* Moore, 1858	Apr	R	Open trail, forest stream	0.002
195.	26B	Pallas' Sailer	*Neptis sappho astola* Moore, 1872	Jan–Dec	vF	Forest streams, riversides, open trails, grasslands	1.276 (mixed with *N. hylas*)
196.	26C	Creamy Sailer	*Neptis soma butleri* Eliot, 1969	Mar–Oct	F	Forest streams, open trails, riversides	0.149
197.	26D	Common Lascar	*Pantoporia hordonia hordonia* (Stoll, [1790])	Jan–Dec	vF	Forest streams, riversides, open trails	1.170
198.	26E	Extra Lascar	*Pantoporia sandaka davidsoni* Eliot, 1969	Jan–Dec	vF	Forest streams, riversides, open trails	1.064
199.	26F	Short‐Banded Sailer	*Phaedyma columella ophiana* (Moore, 1872)	Jan–Dec	F	Forest streams, riversides, open trails, flowers	0.085
200.	26G	Common Earl	*Tanaecia julii appiades* (Ménétriés, 1857)	Mar–Dec	vF	Forest streams, riversides, open/shady trails	1.064
*Subfamily: Heliconiinae*							
201.	26H	Indian Fritillary	*Argynnis hyperbius hyperbius* (Linnaeus, 1763)	Mar–Jan	F	Flowers	0.425
202.	27A	Rustic	*Cupha erymanthis lotis* (Sulzer, 1776)	Mar–Oct	F	Forest streams	0.160
203.	27B	Common Leopard	*Phalanta phalantha phalantha* (Drury, [1773])	Jan–Dec	vF	Flowers	0.745
*Subfamily: Apaturinae*							
204.	27C	Circe	*Hestinalis nama nama* (Doubleday, 1844)	Apr	fR	Riversides	0.003
205.	27D	Eastern Courtier	*Sephisa chandra chandra* (Moore, [1858])	Apr–Nov	fR	Forest streams, Riversides	0.003
*Subfamily: Cyrestinae*							
206.	27E	Common Maplet	*Chersonesia risa risa* (Doubleday, [1848])	Feb–Dec	vF	Open, shady, forest trails, forest streams, flowers	1.064
207.	27F	Common Map	*Cyrestis thyodamas thyodamas* Boisduval, 1846	Jan–Dec	vF	Open trails, riversides, forest streams, flowers	1.010
208.	27G	Tabby	*Pseudergolis wedah wedah* (Kollar, 1848)	Apr, Aug	fR	Forest streams	0.003
209.	27H	Popinjay	*Stibochiona nicea nicea* (Gray, 1846)	Mar–Oct	F	Forest streams, rarely riversides	0.032
*Subfamily: Nymphalinae*							
210.	28A	Indian Tortoiseshell	*Aglais caschmirensis aesis* Fruhstorfer, 1912	Jan–Oct	F	Open trails, flowers, agricultural fields	0.425
211.	28B	Autumn Leaf	*Doleschallia bisaltide* (Cramer, [1777])	Mar, Aug–Nov	F	Forest streams	0.032
212.	28C	Great Eggfly	*Hypolimnas bolina jacintha* (Drury, 1773)	Mar–Oct	F	Open trails, flowers	0.213
213.	28D	Peacock Pansy	*Junonia almana almana* (Linnaeus, 1758)	Jan–Dec	vF	Open trails, open country, grasslands, flowers	1.170
214.	28E	Grey Pansy	*Junonia atlites atlites* (Linnaeus, 1763)	Jan–Dec	vF	Open trails, open country, grasslands, flowers, riversides	1.170
215.	28F	Chocolate Pansy	*Junonia iphita iphita* (Cramer, [1779])	Jan–Dec	vF	Open trails, open country, grasslands, flowers, forest streams, riversides	1.915
216.	28G	Lemon Pansy	*Junonia lemonias persicaria* (Fruhstorfer, 1912)	Jan–Dec	vF	Open trails, open country, grasslands, flowers, forest streams, riversides	1.702
217.	28H	Blue Pansy	*Junonia orithya ocyale* Hübner, [1819]	Jan–Dec	F	Open trails, riversides, grasslands, flowers	0.425
218.	29A	Orange Oakleaf	*Kallima inachus inachus* (Boisduval, 1846)	Mar–Dec	vF	Forest trails, forest, forest streams, riversides	0.638
219.	29B	Blue Admiral	*Kaniska canace canace* (Linnaeus, 1763)	Mar–Apr, Dec	fR	Riversides, forest streams	0.004
220.	29C	Spotted Jester	*Symbrenthia hypselis cotanda* Moore, [1875]	Mar–Nov	F	Riversides, forest streams	0.053
221.	29D	Common Jester	*Symbrenthia lilaea khasiana* Moore, [1875]	Jan–Dec	vF	Open trails, open country, grasslands, flowers, forest streams, riversides	1.276
222.	29E	Painted Lady	*Vanessa cardui* (Linnaeus, 1758)	Jan–Dec	vF	Open trails, open country, grasslands, flowers, riversides, agricultural fields	1.276
223.	29F	Indian Red Admiral	*Vanessa indica indica* (Herbst, 1794)	Jan–Dec	F	Open trails, open country, grasslands, flowers, riversides, agricultural fields	0.425
*Subfamily: Acraeinae*							
224.	29G	Tawny Coster	*Acraea terpsicore* (Linnaeus, 1758)	Sept	vR	Riverside	0.001
225.	29H	Red Lacewing	*Cethosia biblis tisamena* Fruhstorfer, 1912	Mar–Nov	fR	Forest streams, flowers	0.003
226.	30A	Leopard Lacewing	*Cethosia cyane cyane* (Drury, [1773])	Jan–Dec	F	Forest streams, flowers	0.085

A strong positive correlation was found between species richness and average monthly temperature (*r* = 0.831, *p* < 0.01), whereas a weak positive correlation existed between species richness and average monthly precipitation (*r* = 0.363) (Figure 33). The Shannon–Wiener index for the overall study area was 4.71, indicating high diversity. The Pielou's *J* index of 0.87 showed a relatively even distribution of species. Additionally, Margalef's richness index was 19.65, and the effective number of species was 111.24. At the family level, Nymphalidae exhibited the highest diversity, with 86 species, a Shannon–Wiener index of 3.84, Pielou's *J* index of 0.86, and Margalef's richness index of 8.01, indicating the greatest overall diversity and evenness. In contrast, Riodinidae showed the lowest diversity, with only five species, a Shannon–Wiener index of 0.71, Pielou's *J* index of 0.44, and Margalef's richness index of 0.50, reflecting the least diversity and evenness in the study area. Detailed diversity indices for each family are presented in Table 2.

**TABLE 2:** Diversity Metrics for Butterfly Families in Bhorletar—Species richness, Shannon–Wiener index, Pielou's *J* index, effective number of species, and Margalef's richness index for different families and overall study area.

Family	Species richness	Shannon–Wiener index	Pielou's *J* index	Effective number of species	Margalef's richness index
Hesperiidae	46	3.00	0.79	20.21	4.76
Papilionidae	15	2.08	0.77	8.03	1.61
Pieridae	22	2.47	0.78	11.81	2.21
Lycaenidae	52	3.19	0.80	24.38	5.19
Riodinidae	5	0.71	0.44	2.03	0.50
Nymphalidae	86	3.84	0.86	46.55	8.01
Overall	226	4.71	0.87	111.24	19.65

## 5 Discussion

Butterfly diversity in Bhorletar peaks in spring/pre‐monsoon; this season is marked by the production of new tender leaves by host plants and ideal weather conditions, which support the completion of butterfly life cycles. Additionally, several species classified as very rare to fairly rare within the study area, including *Ctenoptilum vasava vasava* (Figure 2H), *Seseria dohertyi dohertyi* (Figure 3D), *Delias belladonna horsfieldi* (Gray, 1831) (Figure 10H), *Arhopala eumolphus eumolphus* (Cramer, 1780) (Figure 13C), *Lethe kansa kansa* (Moore, 1857) (Figure 21G), *Neptis ananta ochracea* (Evans, 1924) (Figure 25B), *N. miah miah* (Moore, 1857) (Figure 25G), and *N. sankara amba* (Moore, 1858) (Figure 26A), are exclusively found during this time. After the spring peak, butterfly diversity experiences a mid‐year slump in June–July/monsoon, followed by a resurgence in August/late‐monsoon and a subsequent decline until January. This bimodal pattern is likely attributed to the monsoon season's unfavorable weather conditions, which hinder butterfly observation owing to flooded rivers, treacherous trails, and potentially a natural decline in butterfly populations caused by natural enemies or the early‐stage phase of multivoltine species' life cycles. In contrast, winter (December–February) represents the lowest point of butterfly diversity, characterized by parched vegetation, dwindling forest streams, and shallow rivers. The prevailing foggy conditions and limited sunlight further exacerbate this decline. Similar annual bimodal patterns in species diversity have been reported in multiple insect groups (Frith and Frith 1985; Plant et al. 2018; Hernández‐Ortiz et al. 2022), including butterflies (Takagi and Miyashita 2015; Gupta, Tiwari, and Diwakar 2019; Naik et al. 2022; Albu and Albu 2023; Sapkota et al. 2024). The Shannon–Wiener index (*H*′ = 4.71), Pielou's evenness index (*J*′ = 0.87), and Margalef's richness index (*D* = 19.65) align with the findings of Miya et al. (2021), Neupane and Miya (2021), and Subedi et al. (2021), who conducted similar studies in neighboring districts during the same years as this survey; however, our values are generally higher.

In this study, Nymphalidae exhibited the highest scores across all diversity indices, primarily attributed to their high global species richness (Hoskins 2015; Catalogue of Life 2024), also observed in Nepal (Smith 2011a). This finding is further supported by their robust flight capabilities and high dispersal capacity (Flockhart et al. 2017; Suchan et al. 2024) along with wide ecological adaptability (Wahlberg et al. 2009). In contrast, the low diversity indices of Riodinidae can be attributed to their relatively low species richness in Nepal (Smith 2011a) compared to other butterfly families. Species such as *Papilio demoleus demoleus* Linnaeus, 1758 (Figure 8E), *Pieris canidia indica* Evans, 1926 (Figure 11H), and *Melanitis leda leda* (Linnaeus, 1758) (Figure 22A), known to be pests of citrus (Rao 2015; Kolosova and Bolotov 2020), crucifers (Chen et al. 2004; Hwang, Liu, and Shen 2008), and paddy (Singh and Sharma 2018; Imtienla et al. 2024), respectively, were commonly observed; however, no significant threats to these crops were evident, suggesting that these species, although present, did not pose a substantial risk to agricultural productivity. To our knowledge, none of the recorded species are currently listed as endangered or protected; however, this may be attributed to a lack of comprehensive assessments and updates on their conservation status. In terms of the correlations among species richness, temperature, and precipitation, a strong positive relationship between species richness and average monthly temperature has been previously observed in ectothermic species, such as butterflies (Turner, Gatehouse, and Corey 1987; Lazarina et al. 2023). In contrast, the weak correlation between species richness and average monthly precipitation may be attributed to the concentration of rainfall during certain months (April–September) and the absence of precipitation during certain months (January, March, October, November, and December), as well as the limitations of this correlation calculation in accounting for other moisture‐related factors, such as humidity and the influence of rivers and streams.

The discovery of several rarely seen species in Nepal further underscores the remarkable butterfly diversity in Bhorletar. Despite the small survey area and limited elevational range, the study resulted in an impressive 226 butterfly species–representing approximately 33% of the total butterfly fauna in Nepal (Van der Poel and Smetacek 2022) and approximately 53% of the midland species (Smith 2011a). The survey area likely benefited from its location within the ecotone between lowland and high hills. The entire area of Bhorletar could have as many as 250 butterfly species. With the establishment of dedicated butterfly puddling grounds and the conservation and enrichment of its natural habitats, Bhorletar has the potential to become a premier butterfly destination. Nepal itself could benefit from establishing butterfly tourism hotspots, similar to the model of Jayanti Village in Buxa Tiger Reserve, West Bengal, India. In conclusion, this study provides valuable insights into the diversity of butterflies in Bhorletar, Nepal, offering a glimpse into the butterfly fauna of the Central Himalayas. Furthermore, it establishes a crucial baseline reference for future research, enabling long‐term monitoring and conservation efforts in this ecologically significant region.

## Author Contributions

**Sajan KC:** conceptualization (lead), data curation (lead), formal analysis (supporting), investigation (lead), methodology (lead), software (supporting), visualization (supporting), writing – original draft (lead), writing – review and editing (equal). **Anisha Sapkota:** conceptualization (supporting), data curation (supporting), formal analysis (lead), investigation (supporting), methodology (supporting), software (lead), visualization (lead), writing – original draft (supporting), writing – review and editing (equal).

## Conflicts of Interest

The authors declare no conflicts of interest.

## Data Availability

Data are archived in Dryad in “Private for peer review” status under doi:10.5061/dryad.ghx3ffbzh, and can be accessed using the following link: https://datadryad.org/stash/share/x8GBcVetKqerc66Jn_JFssK2Fu183RT2eqtaB‐EiEZY.
